# Nearby contours abolish the binocular advantage

**DOI:** 10.1038/s41598-021-96053-9

**Published:** 2021-08-19

**Authors:** Maria Lev, Jian Ding, Uri Polat, Dennis M. Levi

**Affiliations:** 1 School of Optometry and Vision Science, The Mina & Everard Goodman Faculty of Life Sciences, Bar Ilan University, Ramat Gan, Israel; 2 School of Optometry and Helen Wills Neuroscience Institute, University of California Berkeley, Berkeley, CA 94720-2020 USA

**Keywords:** Neuroscience, Psychology

## Abstract

That binocular viewing confers an advantage over monocular viewing for detecting isolated low luminance or low contrast objects, has been known for well over a century; however, the processes involved in combining the images from the two eyes are still not fully understood. Importantly, in natural vision, objects are rarely isolated but appear in context. It is well known that nearby contours can either facilitate or suppress detection, depending on their distance from the target and the global configuration. Here we report that at close distances collinear (but not orthogonal) flanking contours suppress detection more under binocular compared to monocular viewing, thus completely abolishing the binocular advantage, both at threshold and suprathreshold levels. In contrast, more distant flankers facilitate both monocular and binocular detection, preserving a binocular advantage up to about four times the detection threshold. Our results for monocular and binocular viewing, for threshold contrast discrimination without nearby flankers, can be explained by a gain control model with uncertainty and internal multiplicative noise adding additional constraints on detection. However, in context with nearby flankers, both contrast detection threshold and suprathreshold contrast appearance matching require the addition of both target-to-target and flank-to-target interactions occurring before the site of binocular combination. To test an alternative model, in which the interactions occur *after* the site of binocular combination, we performed a dichoptic contrast matching experiment, with the target presented to one eye, and the flanks to the other eye. The two models make very different predictions for abutting flanks under dichoptic conditions. Interactions after the combination site predict that the perceived contrast of the flanked target will be strongly suppressed, while interactions before the site predict the perceived contrast will be more or less veridical. The data are consistent with the latter model, strongly suggesting that the interactions take place before the site of binocular combination.

## Introduction

An important ritual of certain wedding ceremonies involves the groom showing the bride the double star pair Mizar and Alcor in the handle of the big dipper. Successful sighting of the nearly invisible Alcor portends a successful marriage. For the anxious groom, viewing with two eyes provides a distinct advantage. That binocular viewing confers an advantage over monocular viewing for detecting isolated low luminance or low contrast objects (like Alcor), has been known for well over a century and is ubiquitous^1,2^; however, the processes involved in combining the images from the two eyes are still not fully understood. Importantly, in natural vision, objects are rarely isolated but appear in context.

The superiority of binocular over monocular viewing for detecting near threshold isolated targets, about a factor of 1.5^3^, has been documented and quantified in hundreds of studies (reviewed in Refs.^1–3^). We still do not have a full understanding of how the inputs to the two eyes are combined; however, to account for the complexity of binocular interactions under a broad range of different stimuli and tasks, almost all recent models of binocular combination incorporate dynamic gain control^4–6^. As Blake and Wilson^7^ note in their excellent review, “The evidence moving the field in those directions has come largely from experiments that have measured contrast summation at threshold and suprathreshold levels and contrast masking using dichoptically presented grating patterns…”. Most of these studies have involved isolated stimuli; however, it is well known that nearby contours can modulate detection, depending on their distance from the target and the global configuration. For example, nearby flanking contours in a collinear, but not orthogonal arrangement can strongly suppress target detection, while more distant flanking contours can facilitate detection, both in foveal^8,9^ and perifoveal^10^ vision.

Only a few previous studies have examined how nearby contours modulate the manner in which the two eye’s inputs are combined. For example, Huang et al.^11^, measured foveal contrast detection thresholds for Gabor patches, either in isolation, or with flankers at a target-to-flanker distance of 4°. Their results show that flankers facilitate detection, under both monocular and binocular conditions and reveal the familiar binocular advantage. Interestingly, flankers presented dichoptically did not facilitate detection. They conclude that flanker facilitation must operate “at the earliest stages of cortical processing”. A more recent study^12^ evaluated foveal contrast discrimination with and without co-aligned flankers separated from the target by 2.7°, under both monocular and binocular conditions, flankers lowered thresholds (i.e., facilitated detection), thus preserving the binocular advantage. However, dichoptic flankers elevated thresholds at intermediate pedestal contrast levels when the pedestal was dichoptically presented. Based on their modeling, they concluded “that flankers modulate outputs from spatial filters in the monocular processing stage of contrast gain control”.

Importantly, the effects of nearby contours on binocular combination has not been examined in parafoveal vision, which is highly susceptible to lateral interactions, crowding and surround suppression^13,14^, or in the fovea with flankers that impinge on the target, resulting in suppression of detection^15,16^. In the experiments described here we measured contrast summation at threshold and suprathreshold levels for both isolated targets, and targets with nearby flankers at different distances and in different configurations. Specifically, we measured contrast detection thresholds, contrast matching and contrast discrimination for isolated or flanked targets as a function of target flanker separation and contrast at an eccentricity of 4° or in the fovea. In order to better understand and model the results we also tested binocular summation under dichoptic conditions. The specific tasks, conditions and other details are listed in Table 1.

**Table 1:** Details of the experimental design. *CD* contrast detection, *CM* contrast matching, *SF* spatial Frequency, *2IFC* two interval force choice.

Experiment	Task	Eccentricity	SF	Method	N	Figure
1	CD	4	2	2IFC	6	1a, 1c
2	CD	4	2	2IFC	5	1b, 1d
3	CD	4	4	Yes/No	7	2a, 2c
4	CD	0 fovea	6	2IFC	5	2b, 2d
5	CD Half Bino	4	2	2IFC	3	1b, 1d
6	CD Dichoptic Separation and contrast	4	2	2IFC	3 (separation) 1 (contrast) from Exps. 2 & 5	Not shown 1b
7	Contrast discriminatio n	4	2	2IFC	2	3b, 3c
8	CM and CM dichoptic	4	2	2IFC	6 3	4a–d, 4e

Here we report that, surprisingly, nearby collinear (but not orthogonal) flanking contours completely abolish the binocular advantage. Our results for monocular and binocular viewing, for contrast discrimination thresholds without nearby flankers, can be explained by a gain control model with uncertainty and internal multiplicative noise adding additional constraints on detection. However, in context with closely spaced flankers, both contrast detection threshold and suprathreshold contrast appearance matching require the addition of both target-to-target and flank-to-target interactions occurring before the site of binocular combination.

## Results

### Contrast detection

**Figure 1:**
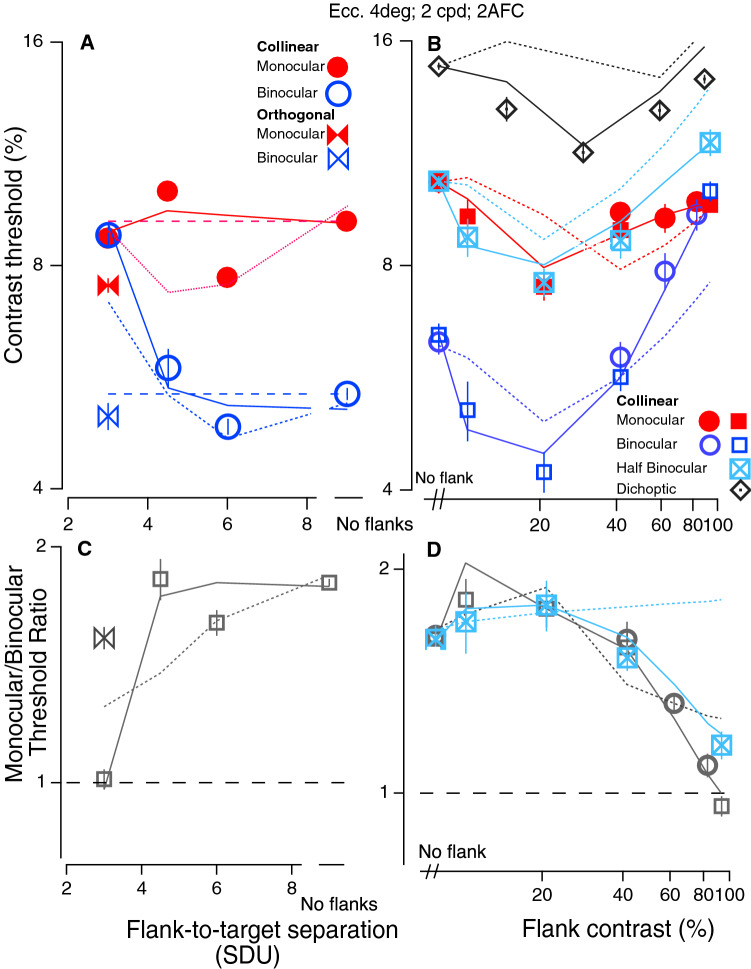
Contrast detection. Contrast detection for binocular (blue) and monocular (red) viewing as a function of flank distance (**A**), with flank contrast = 9.5 × threshold (left; symbols show the mean ± 1se of 6 observers), or as a function of flank contrast. Dashed lines show the unflanked thresholds. (**B**) With abutting flanks (flank-to-target separation = 3SDU; symbols show the mean ± 1se of 5 different observers). Also shown in (**B**) are data under half binocular condition for 3 observers and data under dichoptic conditions for one observer. The solid lines are fits of the GUMI Model, and the dotted lines are fits of the aGUM model (see “Model” for details). (**C**,**D**) Binocular summation factor (monocular/binocular threshold ratio) from (**A**) and (**B**) respectively. Note that the abscissas in (**B**) and (**D**) are plotted on a logarithmic scale.

In the first experiment (Experiment 1) we measured contrast detection thresholds for isolated or flanked Gabor targets as a function of flank-to-target separation (spatial frequency 2 cpd) at an eccentricity of 4°, in either collinear or orthogonal configurations (see Fig. 1).

Figure 1 shows the effect of flanks on contrast detection thresholds for monocular (red symbols) and binocular (blue symbols) viewing as a function of flank-to-target separation (specified in multiples of the Gabor envelope standard deviation, [SDU]) with high contrast flankers (9.5 × threshold (1A), and as a function of flank contrast with abutting flankers (Experiment 2: flank-to-target separation = 3SDU, 1B). For binocular viewing (blue circles), detection is facilitated by flankers at a separation of 6SDU (p = 0.016) and strongly suppressed by abutting collinear (but not orthogonal) flankers (at 3SDU), consistent with previous studies^10,17^ in parafoveal viewing. Interestingly, for monocular viewing, there is similar facilitation (p = 0.005 at 6SDU), but no suppression [monocular threshold elevation at 3SDU is not significantly different from baseline (p = 0.058)].

Importantly, with abutting collinear flankers at the highest flanker contrast level (9.5 × threshold), under these conditions, the monocular and binocular thresholds are essentially identical, and binocular summation (Fig. 1C,D) is abolished. However, at larger separations (1A) and lower contrast levels (1B), they diverge, with binocular thresholds lower than monocular thresholds, consistent with the ubiquitous advantage of binocular viewing of low contrast isolated targets (between a factor of √2 and 2^3^ that has been known for well over a century (for reviews see Refs.^1,2^. Interestingly, abutting orthogonal flankers facilitate contrast detection, but they do not reduce the binocular advantage (bowties in the two left panels).

**Figure 2:**
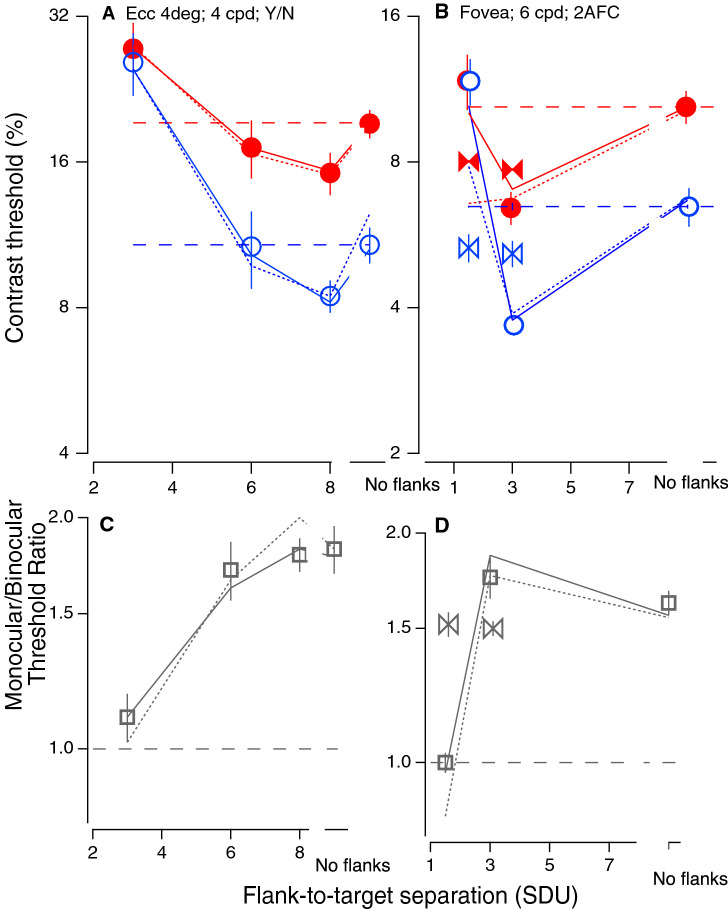
Contrast detection. Contrast thresholds for binocular (blue) and monocular (red) viewing as a function of flank distance (**A**) at eccentricity of 4°, with a different spatial frequency (SF 4 cpd) using a different psychophysical paradigm (Y/N), with flank contrast = 9.5 × threshold (symbols show the mean ± 1se of 7 observers). Dashed lines show the unflanked thresholds. (**B**) Foveal thresholds (eccentricity of 0°, SF 6 cpd, 2AFC, symbols show the mean ± 1se of 5 observers) with flank contrast = 6 × threshold. The solid lines are fits of the GUMI model and the dotted lines are fits of the aGUM model. (**C**,**D**) Binocular summation factor (monocular/binocular threshold ratio) from (**A**) and (**B**) respectively. Dashed lines indicate no summation.

We have replicated this difference between monocular and binocular suppression with a different paradigm and observers, and it raises the intriguing question of whether monocular and binocular interactions are processed by the same network. Specifically, to assess whether this surprising failure of binocular summation with closely spaced collinear flankers occurs under different conditions, we recruited new observers and tested them at the same eccentricity with a different spatial frequency and psychophysical paradigm (Experiment 3: Ecc 4 degrees; SF 4 cpd ; N = 7; Yes/No—Fig. 2A,C), and, in the fovea (Experiment 4: 0 Ecc degrees; 6 cpd; N = 5; 2 AFC—see “Methods”—Fig. 2B,D), in order to see whether these results are due to peripheral crowding . Figure 2A,C essentially replicate the results of the initial experiment. However, with foveal viewing (Experiment 4: Ecc 0 degrees; SF 6 cpd; N = 5; 2 AFC, Fig. 2B), binocular summation is evident with abutting flankers (at a flank-to- target separation of 3SDU). Rather, the foveal data appear to be a scaled version of the perifoveal results, consistent with previous studies^10,18^, with the failure of binocular summation occurring at a smaller separation (1.5 SDU), where the target and flankers partially overlap.

To examine this failure of binocular summation in greater detail, we conducted additional detection experiments. First, we replicated the data of Fig. 1B with three observers (red and blue squares) and added two additional conditions: half binocular (Experiment 5) and dichoptic (Experiment 6). For the half binocular condition^6^ flankers are always binocular (i.e., presented to both eyes), while the target is either monocular or binocular. This has the advantage that the number of eyes stimulated by the mask is held constant, and only the number of eyes stimulated by the target changes across conditions. Under half binocular conditions (light blue squares with Xs in Fig. 1B,D), as with the other conditions, binocular summation is sharply reduced with high contrast (c = 90%) abutting flankers. Second, we tested one of 3 observers under dichoptic conditions (target in one eye and flanks in the other—gray diamonds in Fig. 1B). Note that as flank contrast increases, threshold first drops (i.e., contrast sensitivity increases) and then increases again—reminiscent of the well-known “dipper” function for contrast discrimination^19^. This dichoptic facilitation has been previously reported^6,20,21^ and has important implications for understanding the mechanisms that underlie the effects of nearby flankers.

**Figure 3:**
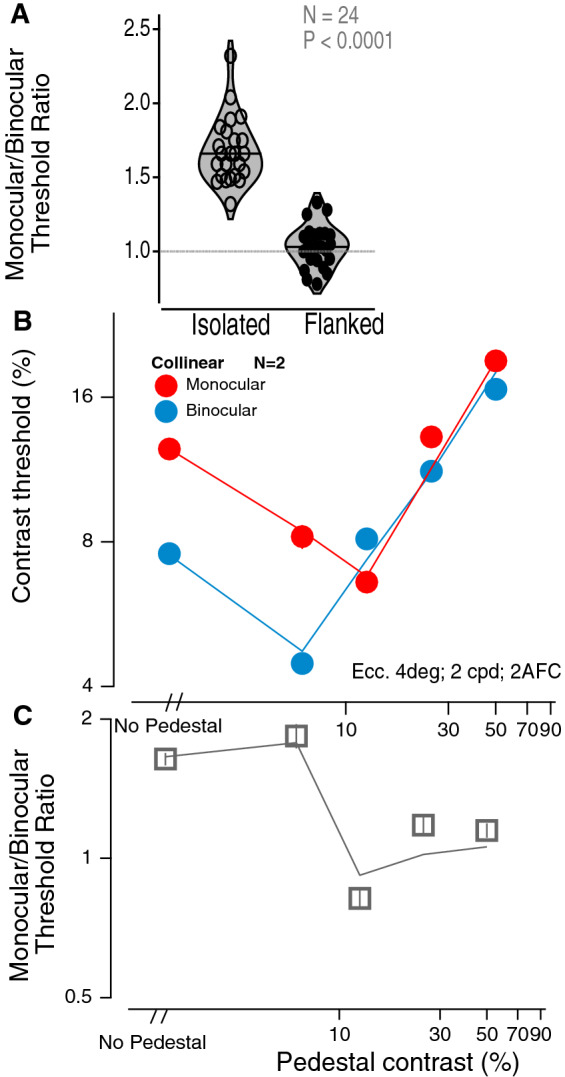
(**A**) The violin plot shows the ratio of monocular to binocular thresholds for 24 independent paired measures (from 16 different observers from experiments 1–5) for isolated vs. closely spaced flanks). The binocular advantage is significantly greater for isolated than for closely flanked stimuli (P < 0.0001). (**B**) Contrast discrimination threshold vs. Pedestal (superimposed flank) contrast. Data from 2 observers. The solid lines are the Ding–Sperling gain control model with uncertainty and multiplicative noise (GUM model: Fig. 5B). (**C**) The monocular:binocular threshold ratio as a function of pedestal contrast.

The absence of a binocular advantage with closely spaced flankers can be clearly seen in the violin plot (Fig. 3A), which shows the ratio of monocular to binocular thresholds for 24 independent paired measures (from 5 experiments and 16 different observers). Similar to previous work^3^, the monocular:binocular threshold ratio for unflanked thresholds is ≈ 1.66 (1.47–2.02); however, for closely spaced flanks it is 1.03 (0.82–1.27). The binocular advantage is significantly greater for isolated than for closely flanked stimuli (P < 0.0001).

### Contrast discrimination

It is well known that a high contrast (superimposed) pedestal abolishes the binocular advantage^22,23^, and we have replicated this result with target and flanks superimposed (Experiment 7; separation = 0; Fig. 3B,C). For pedestal contrasts greater than ≈ 12 percent, the binocular advantage is greatly reduced. This raises the question: do nearby flankers simply act as a (weak) pedestal? Chen and Tyler^24^ argued that a weak pedestal type of model was not compatible with their contrast discrimination data in the presence of nearby flankers. Pedestal models operate through gain control. A simple weak pedestal model operates by combining the target and partial flanker signals from one eye, and these operate together to gain-control the other eye. Our modeling shows that while the Ding–Sperling gain control model with uncertainty and multiplicative noise (GUM model: Fig. 5B) provides a good fit to the pedestal data (lines in Fig. 3B,C), the alternative GUM model (aGUM, see Supplementary Information C) including an equivalent weak pedestal from flanks, does not provide a good fit to our contrast detection threshold data with abutting flankers (dotted lines in Figs. 1 and 2). Adding flank-target interactions to GUM (GUMI model: Fig. 5E) significantly improved model fitting performance (solid lines in Figs. 1 and 2, see Supplementary Information C for model comparison of GUMI vs. aGUM with model fitting statistics).

**Figure 4:**
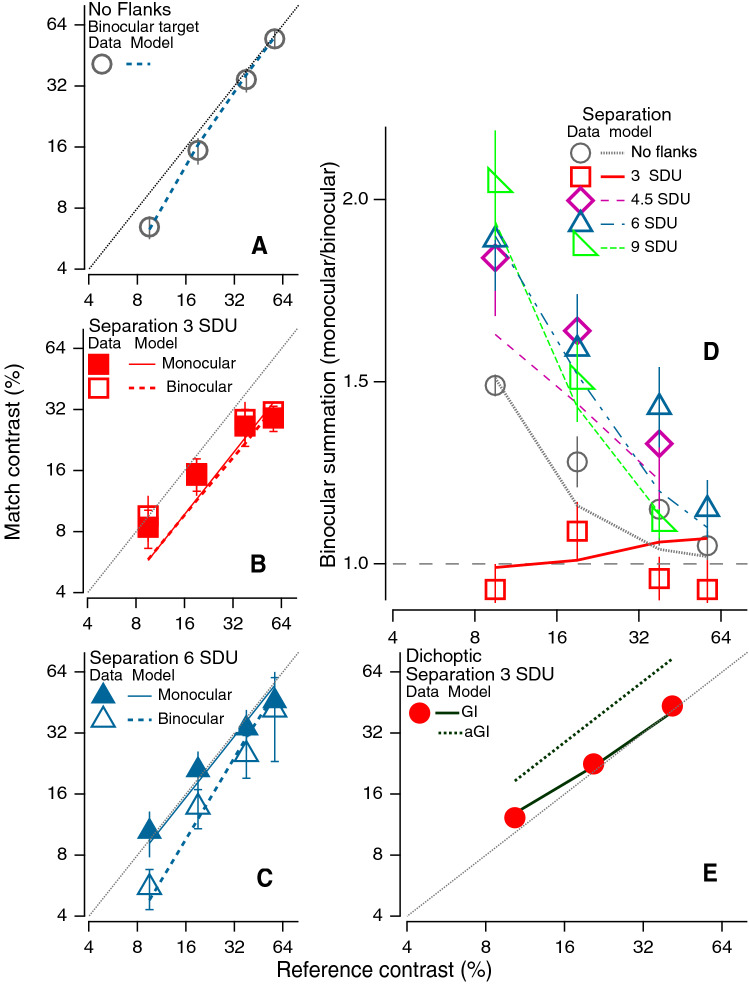
Binocular and monocular matching contrast as a function of reference contrast for a monocular isolated Gabor test patch, as a function of the contrast of an isolated monocular reference (**A**), and for flanked Gabor test patch, presented either monocularly or binocularly (**B** and **C** panels). (**D**) Binocular summation (monocular/binocular contrast ratio) as a function of reference contrast. The flank contrast was fixed at 9.5 × target detection threshold, and the flank-target separation varied, as shown by the different symbols. The lines in panels (**A**–**D**) are the fits of GI model. (**E**) Dichoptic matching (target in one eye, abutting flankers in the other). Data are average data for 3 observers. The model predictions are shown by the thick dotted line (aGI) and the thick solid line (GI).

A superimposed oblique grating or plaid also abolishes the binocular benefit^25^; however, the effects of masking for cross-oriented and pedestal masks are quite different^26,27^. Meese et al.^25^ modeled their results with an architecture based on interactions across orientation and spatial frequency; however their gain control model had a single stage. Our modeling shows that adding a second stage of gain-control of gain-control significantly improved the fit to the data, and our contrast matching with dichoptic flankers (Fig. 4E) provides direct evidence of gain-control of gain-control: flanker's suppression is decreased when target's contrast increases.

Since stimuli in the real world are generally above threshold, next, we performed a contrast matching experiment (Experiment 8, see Table 1, “Methods”) in order to better understand the role of context over a wide range of contrast levels. The observer’s task was to match the perceived contrast of an isolated Gabor patch (the reference) presented to one eye, to that of a flanked Gabor test patch, presented either monocularly or binocularly (lower 2 panels of Fig. 4). Fig. 4A shows matching data for an isolated target viewed with both eyes (ordinate) as a function of the contrast of the monocular reference (abscissa). Consistent with previous work^3,4,6,23,28–30^ the data at the lowest reference contrast falls well below the dotted unity line, indicating binocular summation for near threshold stimuli, and no summation at high contrast levels. In contrast, with abutting collinear flankers (Fig. 4B), there is no evidence of binocular summation (open square and solid squares are not significantly different), and for both binocular and monocular targets, there appears to be facilitation at the highest contrast levels (i.e., both open and solid squares fall below the unity line).

**Figure 5:**
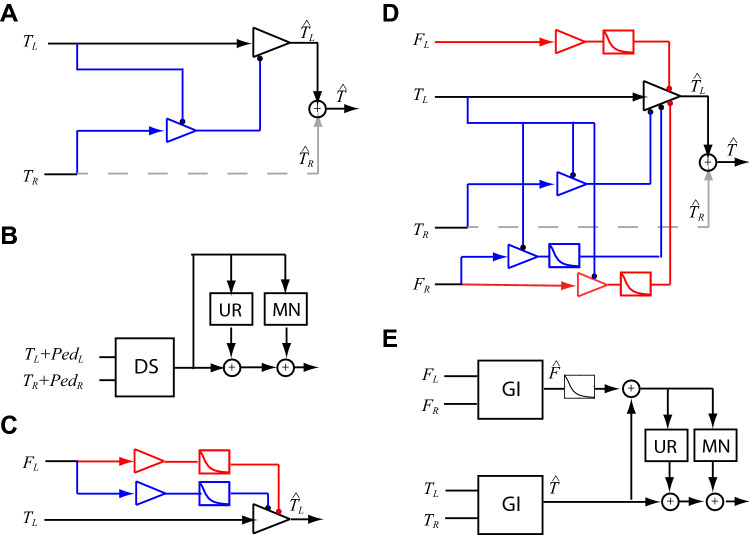
Models. (**A**) DS model: A gain-control model of binocular combination^4^, for target only or target + pedestal condition. Only half of the model is shown here, the other half is symmetric. (**B**) GUM model: DS gain-control model with stimulus-induced uncertainty reduction (UR) and multiplicative noise (MN). (**C**) A model with monocular flanker gain-control (blue) and gain-enhancement (red) of target. (**D**) GI model: DS gain-control model with flanker-target interactions. This is the best fitting model to the matching data (lines in Fig. 4**E**).

The left panels in Fig. 4 show both the binocular and monocular contrast matching (the same data used to compute the binocular summation in the right panel) separately as a function of reference contrast. A *monocular* match contrast below the unity line (gray dotted line) indicates monocular flank facilitation, and a monocular match contrast above the 1:1 line indicates monocular flank suppression. We observed monocular flank facilitation with abutting flanks at high contrast levels (left center panel), but no flank suppression under any condition. A *binocular* match contrast below the monocular match indicates binocular summation. This can be clearly seen for the isolated target only condition (left top panel) where the binocular match contrast falls below the unity line. Interestingly, with abutting collinear flankers (left center panel), binocular summation is eliminated over the entire range of contrasts tested (i.e., the open and solid squares overlap). Increasing the flank to target separation (Fig. 4C for a separation of 6 SDU, and data at 4.5 and 9 SDU shown in Fig. 4D) there is binocular summation and possibly interocular flank facilitation. The Ding–Sperling gain control model with flanker-target interactions (GI model: Fig. 5D) provides a reasonable fit to the contrast matching data for all contrast matching conditions tested (lines in Fig. 4). Including uncertainty and multiplicative noise to the GI model, the GUMI model (Fig. 5E) can also predict the detection data (solid lines in Figs. 1 and 2).

Figure 4D shows binocular summation (the ratio of monocular to binocular matching contrast) as a function of the reference contrast. Each symbol represents a different flanker separation (from the left column, plus additional flanker separations). The target only condition (i.e. for an isolated target viewed binocularly as a function of the contrast of the monocular reference), is consistent with previous work showing binocular summation at low contrast levels but not at high. However, with abutting flankers (flank to target separation = 3SDU) binocular summation is completely abolished at all contrast levels. Surprisingly, with increased separation (Fig. 4D), binocular summation is enhanced, approaching a factor of two! This provides new evidence for interocular gain-enhancement at suprathreshold levels, consistent with a binocular combination model for normal vision^31^, and revealed experimentally in abnormal binocular combination^28^, and later confirmed in normal binocular orientation combination^35^. Because of masking from stronger interocular gain-control, the interocular gain-enhancement is seldom observed under normal viewing condition^28,31^. However, in the present study, because interocular gain-control decayed more quickly than gain-enhancement when increasing flanker-target separation, our binocular summation data shows the evidence at a large separation distance, which can be explain by the GI model (Fig. 5D) including distance weighting functions with different distance decay rates for interocular gain-control and gain-enhancement. Our modeling (Table 2) showed that the flank interocular gain-enhancement occurs before binocular summation, consistent with the previous studies^28,31^, and needs to be gain-controlled from the target. Increasing target contrast decreases the flank interocular gain-enhancement, and therefore decreases the binocular summation factor (Fig. 4D).

**Table 2:** Model comparison.

	Features	*Np*	ν	Average			
				*χ*^2^	*χ*^2^/*v*	AICc	Likelihood
AM 1 (DS)	Without any flank-target interactions	**1**	49	320.3	6.54	97.1	0.00%
AM 2 (wPed)	Inputting a flanker to the DS model as a weak pedestal	**2**	48	319.2	6.65	99.2	0.00%
AM 3	1. With monocular flank’s gain-control 2. Without any interocular flank’s interactions	**2**	48	320.3	6.67	99.4	0.00%
AM 4	1. With monocular flank’s gain-enhancement 2. Without any interocular flank’s interactions	**2**	48	200.5	4.18	76.0	0.00%
AM 5	1. With monocular flank’s gain-control and gain-enhancement 2. Without any interocular flank’s interactions	**3**	47	198.9	4.23	77.9	0.00%
AM 6	1. With monocular flank’s gain-enhancement 2. With interocular flank’s gain-control and the gain-control of gain-control	**3**	47	120.0	2.55	52.7	0.00%
AM 7	1. With monocular flank’s gain-enhancement 2. With interocular flank’s gain-enhancement and the gain-control of gain-enhancement	**3**	47	200.5	4.27	78.3	0.00%
AM 8	1. With monocular flank’s gain-enhancement 2. With interocular flank’s gain-control but without the gain-control of gain-control 3. With interocular flank’s gain-enhancement but without the gain-control of gain-enhancement	**5**	45	93.4	2.08	45.2	0.00%
AM 9	1. With monocular flank’s gain-enhancement 2. With interocular flank’s gain-control and the gain-control of gain-control 3. With interocular flank’s gain-enhancement but without the gain-control of gain-enhancement	**5**	45	70.1	1.56	30.9	0.32%
AM 10	1. With monocular flank’s gain-enhancement 2. With interocular flank’s gain-control but without the gain-control of gain-control 3. With interocular flank’s gain-enhancement and the gain-control of gain-enhancement	**5**	45	121.5	2.70	58.4	0.00%
AM 11	1. With monocular flank’s gain-enhancement 2. With interocular flank’s gain-control and the gain-control of gain-control 3. With interocular flank’s gain-enhancement and the gain-control of gain-enhancement *after the binocular site*	**6**	44	75.2	1.71	37.1	0.01%
AM 12	1. With monocular flank’s gain-enhancement 2. With interocular flank’s gain-control and the gain-control of gain-control *after the binocular site* 3. With interocular flank’s gain-enhancement and the gain-control of gain-enhancement *after the binocular site*	**6**	44	94.0	2.14	48.2	0.00%
AM 13 (aGI)	1. With monocular flank’s gain-enhancement 2. With interocular flank’s gain-control and the gain-control of gain-control *after the binocular site* 3. With interocular flank’s gain-enhancement and the gain-control of gain-enhancement *after the binocular site*	**6**	44	60.4	1.37	26.1	3.55%
AM 14 (GI)	1. With monocular flank’s gain-enhancement 2. With interocular flank’s gain-control and the gain-control of gain-control 3. With interocular flank’s gain-enhancement and the gain-control of gain-enhancement	**6**	44	52.9	1.20	19.5	96.12%

*Np* the number of parameters, *ν* degrees of freedom.

## Modeling

To gain a better understanding of the role of context in binocular combination, we fit our contrast matching data and detection with a gain control model^4^ that includes interocular contrast gain-enhancement and a binocular fusion mechanism^28^, multiplicative internal noise^32^, and interocular luminance gain-control^33^. These models have been shown to predict binocular phase and contrast combination^4,28,34^, binocular contrast discrimination^32^, binocular orientation combination^35^, binocular luminance combination^33^, and stereoscopic depth and cyclopean contrast perception^36–38^ (we note that two latter models differ in their details). An important feature of the model is the inclusion of target-flank distance weighting functions for flanker-induced uncertainty reduction (UR) and multiplicative noise (MN), and for monocular and interocular flanker gain-control, and monocular and interocular flanker gain-enhancement (Fig. 5; see “Methods” for details and model parameters). The distance weighting on the flanker’s gain-control of the target limits the total responses of both target and flanker in a local area within a normal operational range when they are abutting, while their responses are processed individually when they are separated in distance.

The DS gain-control model (Fig. 5A), originally proposed by Ding and Sperling (4), was developed to explain contrast discrimination (Fig. 3B,C) by including a linear contrast transducer and stimulus-induced UR (Refs.^39–42^), and MN^32^ (GUM model: Fig. 5B). Although GUM predicts some features of contrast detection in context with neighbor flankers (dotted lines in Figs. 1 and 2) by assuming that a flanker performs as an equivalent weak pedestal to influence contrast detection (aGUM: alternative GUM, see Supplementary Information A and C), the model fits fall far from the data, especially in Fig. 1 with data under multiple experimental conditions. More importantly, the weak pedestal assumption of a flanker is not consistent with our daily experience in suprathreshold contrast perception. Indeed, the DS model with inputs combining target and weak pedestal of flanker (wPed model or AM2, see Supplementary Fig. A1 A in Supplementary Information A) fails to explain our contrast matching data—the model fitting performance was even worse than the original DS model without any flanker-target interaction (see Table 2).

To explain suprathreshold contrast perception in context with neighboring flankers, we developed a series of models by adding flanker-target interactions to the DS gain-control model (see Supplementary Information A). Among them, the best fitting gain-control model with flanker-target interactions (GI: Fig. 5D) provides a reasonable fit (*χ*^2^ = 1.20;) to our contrast matching data (Fig. 4). After adding stimulus-induced UR and MN to GI, GUMI (Fig. 5E) accurately predicts contrast detection data (the solid lines in Figs. 1 and 2). Please note that, after removing the flankers (flanker contrast = 0), GUMI becomes GUM (Fig. 5B), which can predict contrast discrimination (Fig. 3B,C) as discussed above. GUMI provides a unified explanation of all our data of contrast matching, detection, and discrimination.

Below the detection threshold, observers are uncertain about which visual channels to monitor. Adding a stimulus reduces such uncertainty, and therefore, reduces the internal noise equivalently^39^. This stimulus-induce UR provides a unified explanation for the contrast detection/discrimination facilitation induced by a low-contrast pedestal (the dip in Fig. 3B), a low-contrast abutting flanker (the dip in Fig. 1B), and a high-contrast distant flanker (the dip in Figs. 1A, 2A,B). The reduction in uncertainty not only results in facilitation, but also predicts a lower psychometric function slope. This has been shown to occur both with low contrast pedestals^6^, but also with nearby flankers^16^. Our control experiment with dichoptic flanker (target and flankers presented to different eyes; black diamonds in Fig. 1B) also shows similar contrast detection facilitation, direct evidence of UR, which may not be explained by either an accelerating non-linear contrast transducer or monocular gain modulation. The GUMI model with fixed parameters (Supplementary Table C2 in Supplementary Information C: best fits to monocular, binocular and half binocular data in Fig. 1) correctly predicted the dichoptic data in Fig. 1. (We note that based on their experiments and modeling, Meese and Summers^43,44^ rejected uncertainty reduction as an explanation for facilitation).

On the other hand, adding a stimulus (pedestal or flankers) may also increase multiplicative internal noise (e.g., MN), which gives reasonable predictions for flanker/pedestal masking effects (the handles in Figs. 1, 2, 3). In a previous study^32^ of contrast discrimination with pedestals, we assumed an accelerating non-linear contrast transducer to explain the facilitation by low-contrast pedestals, and MN to explain the masking effect of high-contrast pedestals. The model, with early internal noise (before the binocular site), provided a better fit to the data than one with late noise (after the binocular site) because the model with early noise has a larger signal-to-noise ratio than the model with late noise, resulting in a binocular advantage that was more consistent with the data. In the present study, we tested multiple conditions, however, the data in each condition were not sufficient to differentiate between the models with early versus late noise. For simplicity, late internal noise was added to the model after the binocular site.

Our gain-control model makes several predictions: (1) binocular summation = 2 when the gain-control is close to zero at very low stimulus contrast levels, (2) binocular summation = 1 when the gain-control is high, at high stimulus contrast levels, and (3) binocular summation ~ 1.5 when the gain-control is near the contrast threshold^4,28^. As shown in Fig. 4 (right panel), for the target only condition (open circles), the summation factor decreases from ~ 1.5 to ~ 1 when the target contrast increased from threshold to six times threshold, consistent with the model prediction (gray dotted line). (4) Abutting flankers (red squares), exert additional interocular gain-control of the target, such that the total gain-control eliminates binocular summation, reducing the summation factor to ~ 1 even when the target contrast was at the threshold level. (5) Further increasing flank-target separation (diamonds and triangles), increases the summation factor beyond that for an isolated target, showing interocular facilitation of the target by the flankers. This flank-to-target interocular gain-enhancement counter-balances both target-to-target and flank-to-target interocular gain-controls, resulting in greater binocular summation.

Please note that these flank-to-target monocular and interocular gain-enhancements are not sufficient to explain the facilitation of contrast detection *at the threshold level* in Figs. 1 and 2. An additional assumption of internal noise reduction (e.g., flank-induced uncertainty reduction) is necessary for the model to explain these detection facilitations. In the literature, the flank-induced detection/discrimination facilitation is well documented either within a channel (see Fig. 3B: contrast discrimination with pedestal) or between channels^45–48^ (also see Figs. 1 and 2). Meese and Baker^46^ proposed a model with gain enhancement located after binocular combination for contrast detection facilitation. With no constraints from contrast matching at suprathreshold levels, their models^45,46^ successfully explained the contrast detection facilitation induced by cross-orientation masks with a single free parameter for gain enhancement across all spatiotemporal conditions and eyes. However, it is not clear whether their observers would also perceive a higher contrast grating with a cross-orientation mask at suprathreshold levels. The gain enhancement in our Model GI (Fig. 5D) is different from the gain enhancement in the model of Meese and Baker^46^: (1) the former was proposed to explain contrast matching at suprathreshold levels, in which the contrast gain is relatively more involved, while the latter was proposed to explain contrast detection facilitation at threshold levels, where uncertainty reduction might have contributed; (2) the former was placed before binocular summation, which significantly improved model fitting performance in binocular combination of phase^28,31^, orientation^35^, and contrast^28,31^ (also see Table 2), while the latter was placed after binocular summation, which has no effect on binocularly combined phase and orientation, and has equal effect with monocular and dichoptic masks (we note that while this is true in terms of model implementation, it is not operationally true because of the different within- and cross-eye suppressions weights within that model); (3) the former receives gain-control from the target, which significantly improved model fitting performance in binocular combination of phase^28,31^, orientation^35^, and contrast^28,31^ (also see Table 2), while the latter receives no gain-control from the target. As pointed out in Ding et al.^31^, the balance of gain-control and gain-enhancement may play an important role in constant contrast perception in binocular vision when contrast varies in the two eyes. Without gain-enhancement, the model with only gain-control would predict Fechner’s paradox^31^: the binocularly combined contrast is weaker than the stronger contrast of the two eyes when the other eye is presented with very low contrast, which violates the contrast constancy.

We also tested the twin summation model^12,20^. The model has seven parameters including four power parameters for calculating the binocular excitation and inhibition responses based on target and flank contrasts in the two eyes. However, the four power parameters were strongly correlated with each other when fitting our contrast matching data (Fig. 4), which resulted in a singularity. More importantly, the twin summation model does not have distance parameters, and, it has only been tested on contrast detection and discrimination when the flank-target distance was fixed (12). Logically, without upgrading, this model has little chance to explain our data with multiple flank-target distances.

We tested 14 models with different combinations of flank-target interactions (details see Supplementary Information A) and found that GI (Fig. 5D) is the best, with relative likelihood of 96%. We compared these 14 alternative models (AMs) based on the Akaike Information Criterion (AIC), a measure of the relative goodness of fit of a statistical model developed by Akaike^49^ (for details see Supplementary Information B). For a set of models, the one with the lowest AICc score is most likely to be the best model of those considered. We compared 14 AMs with their fitting chi-square (*χ*^2^), reduced chi-square (*χ*^2^/*v*), AICc scores and relative likelihoods (Table 2). Without any flank-target interaction, the DS model (AM 1) fit the data very poorly with 0% likelihood. Inputting a flanker to the DS model as a weak pedestal (AM2 or wPed) even worsens the model performance.

Adding monocular flank’s gain-control (AM 3 or AM 5) has no improvement. Although adding monocular flank’s gain-enhancement (AM 4) improves data fit, the likelihood is still 0%. Interocular interactions are needed to explain the data. However, with only flank interocular gain-control (AM 6) or gain-enhancement (AM 7), the likelihood is still 0%. The model requires both interocular flank gain-control and gain-enhancement (e.g., GI). The model also needs the target’s suppression (gain-control) for both interocular flank’s gain-control and gain-enhancement (e.g., GI). Only with the interactions in the signal path for interocular gain-control (AM 10), interocular gain-enhancement (AM 9), or both (AM 8), the model performance is low with less than 1% likelihood. The three alternative models with interactions after the binocular site have low likelihoods. The model with only gain-enhancement after the binocular site (AM11) has poor performance with 0.01% likelihood. However, one of the two alternative models with both gain-enhancement and gain-control after the binocular site has performance of 3.55% likelihood (AM 13 or aGI: alternative GI). Please note that the interactions after the binocular site affect both monocular and binocular perception (see Supplementary Figs. A4 and A5 for AMs 12 and 13 under binocular and monocular conditions), while interocular interactions before the binocular site only affects binocular perception. Most likely, all interocular interactions occur before the binocular site, e.g., GI with 96.13% likelihood.

To further test the aGI model, we performed a control experiment using dichoptic flanks (Fig. 4E) with the target in one eye and flankers in the other. As shown in Fig. 4E, the apparent contrast of target was suppressed at low contrast by a dichoptic flanker, but suppression from the dichoptic flanker was reduced by increasing reference contrast, consistent with gain-control of gain-control. GI with fixed parameters given by Table 3 (best fits to contrast matching data with monocular and binocular flanks) correctly predicts the data (solid line); the interocular flanker’s gain-control of the target explains the dichoptic flanker suppression and interocular target gain-control of flanker gain-control explains the suppression reduction when increasing target contrast (see Supplementary Fig. A5 C for GI model under dichoptic condition). However, under dichoptic condition, aGI only has flanker’s gain-control of target without target’s gain-control of flanker gain-control (see Supplementary Fig. A5 D for aGI model under dichoptic condition)—resulting in poor predictions with much larger dichoptic suppression and no suppression reduction.

**Table 3:** Model 1 parameters.

Gain-controls and gain-enhancement					Distance weighting functions					
${g}_{c}$	${g}_{e}$	$\gamma$	$\alpha$	$\beta$	${D}_{\mathrm{mfe}}$	${q}_{\mathrm{mfe}}$	${D}_{\mathrm{ifc}}$	${q}_{\mathrm{ifc}}$	${D}_{\mathrm{ife}}$	${q}_{\mathrm{ife}}$
1	1	1.76 ± 0.14	1	0.48 ± 0.27	0	4.16 ± 0.33	0	2.78 ± 0.94	5.60 ± 4.04	2.23 ± 0.29

## Discussion

While the advantage of binocular over monocular vision for detecting isolated low contrast or luminance stimuli has been well documented for over a century, our results show that nearby collinear (but not orthogonal) flanking contours completely abolish the binocular advantage, while surprisingly, more distant flanking contours facilitate both monocular and binocular sensitivity. The failure of binocular summation is largely due to the strong interocular, but weak or absent monocular suppression for abutting stimuli. The results are surprising in part because binocular summation is generally found with low visibility (near threshold) stimuli^1,2^ and the effect of the closely spaced flanking features was to reduce stimulus visibility, which one might have (naively) thought would increase the likelihood of binocular summation.

The failure of binocular summation with close flankers shares certain characteristics with a number of well documented flanking phenomena that modulate the visibility of stimuli, among them, crowding, masking, surround suppression, etc.^13–15,50,51^. For example, it is orientation specific and depends on target-to-flank distance. However, we believe that the effect of close flankers on binocular summation is not likely to be a consequence of crowding, since crowding reduces stimulus identification and discrimination, but not detection thresholds^13,15,50,52,53^. Moreover, the critical spacing (in degrees of visual space) for crowding at given eccentricity is independent of target size^50^, whereas the effects reported here are size dependent (i.e., they vary with the target standard deviation). For example, experiments 1 and 3 were both at the same eccentricity (4°), but with different target sizes. For both sizes, the target-to flank spacing producing the suppression of binocular summation was the same in multiples of target size (≈ 3SDU)—i.e., a factor of two different in degrees of visual space (1.2° vs 0.6°).

Gain control is the visual nervous system’s main mechanism for modulating neural responses (both monocular and binocular) in order to stay within their normal operating range. Gain control is evident from retina to cortex and can be implemented via lateral interactions or feedback^54–57^. Indeed, the role of gain control in the relationship between luminance (or contrast) input and perceived brightness (or contrast) was recognized more than 50 years ago^58^. Gain control is a central feature of several models of binocular combination in both animals^59,60^ and humans^4–6,11,12,33,34,61–63^, and a recent optical imaging study of ocular dominance columns in non-human primate V1^64^, has shown that a variant of our model can account for the alterations in interocular balance following short-term monocular deprivation.

Receptive fields (RFs) that share similar response properties are clustered together, forming functional domains. Each cortical site is laterally connected through an extensive network of intrinsic projections known as horizontal connections, mainly in layers 2/3. These tend to be connected along the collinear direction^65^. Malach et al.^66^ reported that “long-range intrinsic connections tended to link the monocular regions of same-eye ocular dominance columns and that binocular domains formed a separate set of connections in area V1; binocular regions were selectively connected among themselves but were not connected to strictly monocular regions, suggesting that they constitute a distinct columnar system”. Collinear flankers modulate the responses of neurons in early visual cortex, resulting in facilitation at low contrast (near threshold) and suppression for high contrast^65^.

Our results for monocular, binocular and dichoptic viewing both at threshold and suprathreshold levels, are captured by a gain control model with both target-to-target and flank-to-target interactions occurring before the site of binocular combination, consistent with previous psychophysical^11,12^, and physiological studies in cat (Refs.^67,68^ and monkey^69^. When the target and high contrast flanks overlap, either physically or within the same receptive fields, the binocular response is high, which drives sublinear integration of the 2 eyes inputs^70^.

In summary, our experiments and modeling confirm and extend previous work^11,12,17,43,44^, over a wide range of conditions (separations, eccentricities and contrast levels) showing that a gain control model with flank-to-target and target-to-flank interactions can also predict the effects of context on monocular and binocular contrast detection and matching, and the complete failure of binocular summation when the separation of the flanking contours is too small.

## Methods

### Participants

A total of 18 adult observers participated in 8 experiments in the study (some participated in more than one experiment, see Table 1 for details; ages from 21 and 38 years). All had visual acuity of 20/20 (LogMar 0) or better in each eye and no more than one-line difference between eyes. The experimental protocol was approved by the internal board of the ethics committee (IRB) of Bar-Ilan University, according to the guidelines and regulations for human subject research. All experimental protocols were performed in accordance with the guidelines provided by the committee approving the experiments. All participants signed an informed consent and received monetary compensation for their time and travel for participating in the study.

### Apparatus

We utilized a customized platform for psychophysical experiments (PSY, Yoram Bonneh). The stimuli consisted of Gabor patches that were displayed using an Eizo, FG-2421, 24″, HD monitor running at 120 Hz, which overcomes display time uniformity issues faced by other LCD monitors and is therefore suited to psychophysics The effective size of the monitor screen was 52 by 30 cm, with a resolution of 1920 × 1080 pixels. The screen was calibrated and Gamma corrected using a luminance meter LS-100 (KONICA MINOLTA).

We used 3D-Vision-2 Wireless Glasses to control the monocular and binocular vision. The consumer version of NVIDIA 3D Vision consists of wireless LCD shuttered glasses that receive an infrared signal from an emitter connected to a PC via a USB cable. The glasses are shuttered at 120 Hz frequency, updating each eye 60 times per second (60 Hz) for a flicker-free stereoscopic experience. An active shutter 3D system involves a technique of displaying stereoscopic 3D images. It operates by presenting each eye’s stimulus in rapid alternation. For monocular viewing one eye’s stimulus is alternated with a mean luminance screen to the other eye, at a rate of 120 Hz. For binocular viewing the left and rights eye’s stimuli are alternated. Across trials, the stimuli and eye (Right, Left, Binocular) were randomly interleaved. Observers were unaware of which eye was being stimulated on any given trial. The background luminance was 50 cd/m^2^ as measured through the shutter goggles. No cross talk was found when using the googles.

## Stimuli and procedures

### Contrast detection (CD, experiments 1–6)

**Figure 6:**
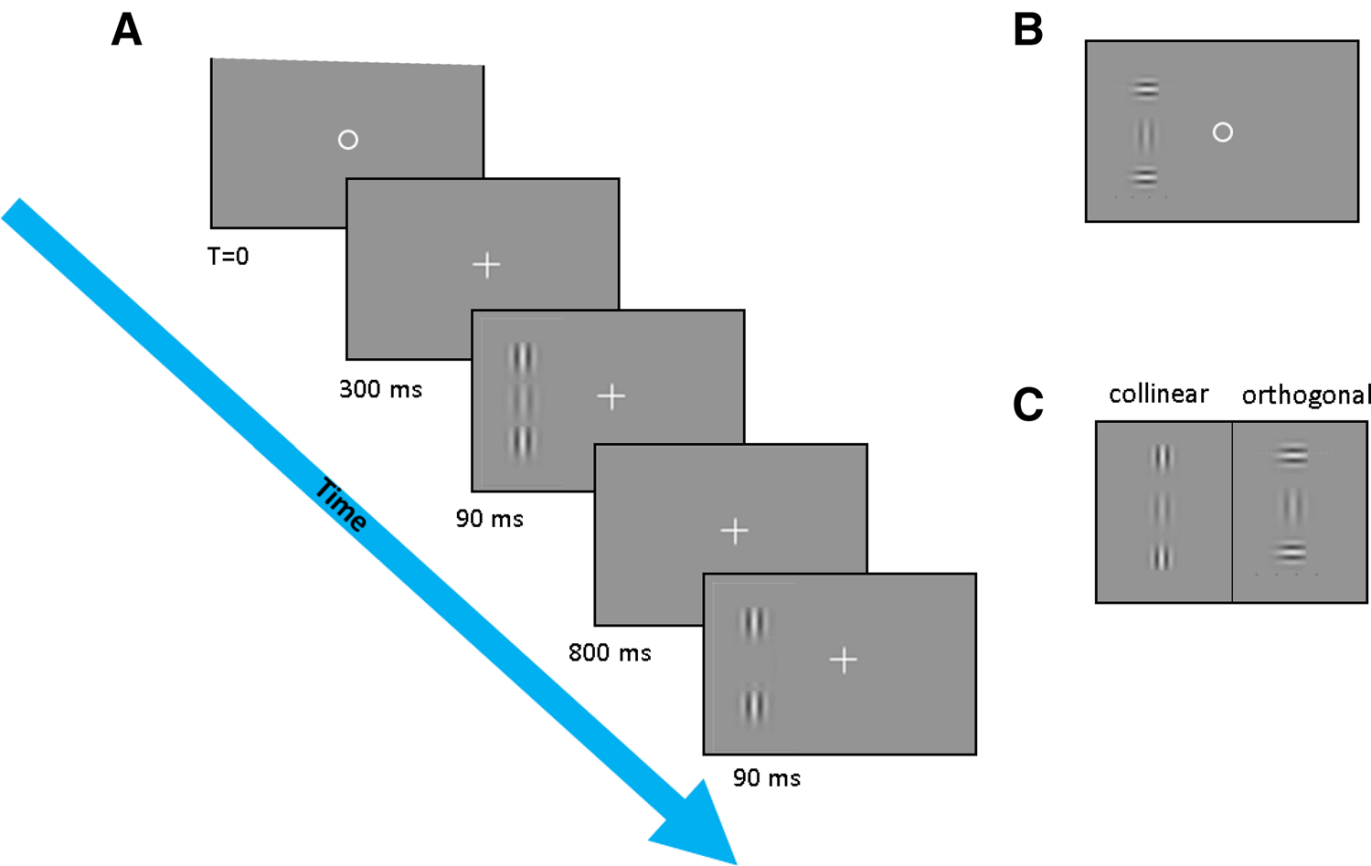
Stimuli. (**A**) Stimuli (collinear) presented at 4° in the periphery. The circle and the crosses indicates the fixation point at the fovea. The temporal sequence of the presentation from the initial trial until it presented either at the left or the right side (as presented here) on the periphery. (**B**) Example of orthogonal stimuli presented at the left side (**C**) two configurations that used in the study. Collinear (left) and orthogonal (right).

In the detection experiments, the viewing distance was 60 cm for the experiments in the periphery and 150 cm for the fovea. We measured contrast detection threshold for a Gabor patches in collinear or orthogonal configurations. The stimuli were briefly presented (90 ms) localized vertical Gabor patches with carrier spatial frequency of 2 and 4 cpd for the periphery and 6 cpd for the fovea (see Fig. 6 and Table 1) and envelope standard deviation of 0.5°, 0.25° and 0.167°, respectively. Contrast thresholds were estimated using the two-interval forced choice (2IFC) paradigm (Yes/No in control exp. 3) via a 3down:1up staircase method, which was shown to converge to 79% correct. In this method, the target contrast is increased by 0.1 log unit (26%), after an erroneous response, and is decreased by the same amount after three consecutive correct responses. Audio feedback is provided to the subject after an incorrect response. There were 8 reversals and the threshold determined from the average of the last 6 reversals.

A visible fixation circle was presented in the center of the screen until the participants pressed the button to start the trial. Then a fixation cross appeared at the center and remained during the trial. To prevent eye movements, the stimuli appeared either at 4° to the right or to the left of the central fixation point, at random. Catch trials were added to ensure foveal fixation on the circle.

The same method was applied for the experiment at the fovea (exp. 4) with the following exceptions: the target and flankers appeared only at the center location without catch trials, and the fixation cross disappeared 300 ms after the beginning of the trial. The flanker contrast was constant at 60%, the target-flank distance was 1.5 and 3 SDU. Flank’s orientation was either collinear or orthogonal configuration. The spatial frequency was 6 cpd.

The order of presentations was mixed-by-trial for right eye, left eye and binocular conditions. Once the subject pressed the button, a 300 ms blank period with fixation until the first stimuli presentation of 90 ms appeared. Both the target and non-target options had the same presentation duration with time intervals of 500 with jitter of 300 ms between them. The target Gabor was presented in only one of the two intervals (the order was randomized). Participants were asked to report which interval contained the target by pressing a mouse button (left for the first interval and right for the second). Across trials, the target presentation was equally distributed between the two intervals. For each condition, monocular and binocular stimuli were randomly interleaved.

Participants were instructed to maintain their fixation at the center of the monitor and to avoid eye movements during the trials (see Fig. 6). Final thresholds are based on average of the left and right sides thresholds obtained at each block, and 4 repetitions at different sessions, thus the data presented in this report are the mean of the 8 separate estimates (280–320 trials).

In experiments 1, 3, 4 (see Table 1) for the periphery 4° and 2 cpd we tested contrast detection threshold for isolated or flanked target. The flank-target’s separations were 3, 4.5, 6 and 9 SDU for the collinear configuration and 3 SDU for the orthogonal configuration. For the 4 cpd, the flank-target’s separations were 3, 6, 8 SDU for the collinear configuration. For the foveal experiment (4) the flank- target’s separations were 1.5 and 3 SDU.

In experiment 2 (see Table 1), we tested the effect of flank’s contrast on the target detection. We used 4 flanker’s contrasts 4, 6, 8 and 9.5 times the target threshold. The flank-target’s separation was 3 SDU. All other methods were as above.

### Effect of flanker’s contrast under half-binocular condition (Experiment 5)

In this experiment we measured the effect of flanker contrast for 3 conditions: monocular, binocular and half-binocular. In the half-binocular condition, the target was presented to one eye (right or left), and the flanks to both eyes. The flank distance was constant, 3 lambda, and flanker’s contrasts was either 1, 2, or 4 times of the target’s threshold or at fixed contrast of 90%. The flanks and the target appeared at an eccentricity of 4 degrees. The tasks were contrast detection and all Methods were the same as experiment 1. Three subjects participated in this experiment.

### Dichoptic contrast detection and matching (Experiment 6)

In this experiment, the reference target was presented to one eye (right), and the flanks to the other (left). The tasks and Methods for contrast detection and contrast matching (were the same as for experiments 1 and 8 respectively). There were 3 subjects who also participated in experiments 1 and 5.

### Effect of flanker contrast under dichoptic condition (Experiment 6)

In this experiment we measured effect of flank contrast for 3 conditions: monocular, binocular and dichoptic (target presented to one eye, and the flanks to the other). Flank’s distance was 3 SDU and flank contrasts was either 1, 2, and 4 times the target’s threshold contrast or at a fixed contrast of 90%. The flanks and the target appeared at eccentricity 4°. The task was contrast detection and all the Methods were the same as experiment 1. There was one subject.

### Contrast discrimination (pedestal) (Experiment 7)

In this experiment we measured contrast discrimination threshold for 2 conditions: monocular and binocular using the 2IFC method. One interval contained only the flanker at the target location (target-flank distance = 0) and the other interval contained flank plus the target. Flank contrast was 1, 2 and 4 times the target’s contrast threshold. The flank and the target appeared at eccentricity 4 degrees with spatial frequency of 2 cpd. The Methods were the same as experiment 1. Two subjects participated in this experiment.

### Contrast matching (Experiment 8)

The stimuli and paradigm are the same as above, (2IFC) with spatial frequency of 2 cpd at eccentricity of 4° and staircase 1 up:1 down with step of 0.05 log unit. The task is to match the isolated target which serve as monocular reference (right eye) with monocular or binocular flanked target. The reference was always presented to right eye and matching performed for monocular (right eye) or for binocular viewing. The reference appeared either in the first or second interval and the subject’s task was to report which interval has higher contrast. The order of presentation was mixed-by-trial (first or second) and to the right or left side of the fixation. There were 4 reference contrasts, 1, 2, 4 and 6 times the contrast threshold. There were 16 reversals (about 35–40 trial per measure) and the threshold determined from the average of last 14 reversals. Final thresholds are based on average of the left- and right-side thresholds of each session and 4 repetitions at different sessions, thus the data point based on 8 measurements (280–320 trials). We measured the contrast matching for both isolated and flanked targets. The flanker-target’s separations were 3, 4.5, 6, 9 SDU for the collinear configuration and 3 SDU for the orthogonal configuration (6 subjects). We also measured the matching under dichoptic condition (3 subjects).

## Model

As shown in Fig. 5A, the DS gain-control model, originally proposed by Ding and Sperling^4,34^, comprises double layers of mutual interocular gain-controls before linear binocular combination. In the layer of signal path (Black in Fig. 5A), the LE’s signal is gain-controlled by the RE, and in the layer of gain-control path (Blue in Fig. 5A), the RE’s gain-control of the LE is gain-controlled by the LE. Figure 5A only shows a half model for LE, and the other half for RE has a symmetric structure. One eye’s gain-control is proportional to the total contrast energy, a weighted summation across all spatial- frequency channels^4,34^, and is also proportional to the mean luminance^33^, i.e., the eye with higher total contrast energy and/or higher mean luminance would give more contribution to the binocularly combined image. In this study, because we only used Gabor patches as stimuli with their mean luminance (constant) as the background luminance, one eye’s gain-control is proportional to the contrast of a Gabor patch presented to that eye.

Let ${T}_{\mathrm{L}}$ and ${T}_{\mathrm{R}}$ be targets’ contrast presenting to the two eyes, and ${G}_{\mathrm{L}}^{\mathrm{itc}}$ and ${G}_{\mathrm{R}}^{\mathrm{itc}}$ be two eyes’ gains after interocular target-target gain-control. In the target only condition, without abutting flanks, the model output is given by,

1 $$\widehat{T}={G}_{\mathrm{L}}^{\mathrm{itc}}{T}_{\mathrm{L}}+{G}_{\mathrm{R}}^{\mathrm{itc}}{T}_{\mathrm{R}}.$$

The two eyes’ gains after gain-controls are given by,

2 $$G_{{\text{L}}}^{{{\text{itc}}}} = \frac{1}{{1 + \frac{{\left( {\frac{{T_{{\text{R}}} }}{{g_{c} }}} \right)^{\gamma } }}{{1 + \alpha^{\gamma } \left( {\frac{{T_{{\text{L}}} }}{{g_{c} }}} \right)^{\gamma } }} }}\quad G_{{\text{R}}}^{{{\text{itc}}}} = \frac{1}{{1 + \frac{{\left( {\frac{{T_{{\text{L}}} }}{{g_{c} }}} \right)^{\gamma } }}{{1 + \alpha^{\gamma } \left( {\frac{{T_{{\text{R}}} }}{{g_{c} }}} \right)^{\gamma } }} }},$$

where ${g}_{c}$ is gain-control threshold and $\alpha$ is the relative gain-control efficiency in the gain-control path (blue) when the gain-control efficiency in the signal path (black) is assumed to be one. Because ${g}_{c}$ is close to monocular contrast detection threshold^32^, in this study, we set ${g}_{c}$ = monocular contrast detection threshold for simplicity, and we also set $\alpha =1$ for fitting contrast matching data. Therefore, the DS model only has one parameter $\gamma$. The model predicts that (1) at small stimulus contrast (${T}_{\mathrm{L}}={T}_{\mathrm{R}}\ll {g}_{c}$), no gain-control occurs (${G}_{\mathrm{L}}^{\mathrm{itc}}={G}_{\mathrm{R}}^{\mathrm{itc}}\approx 1$), resulting in linear binocular summation, i.e., the summation factor = 2; (2) at high stimulus contrast (${T}_{\mathrm{L}}={T}_{\mathrm{R}}\gg {g}_{c}$), the gain-control reaches the maximum, and each eye’s signal is halved before linear combination (${G}_{\mathrm{L}}^{\mathrm{itc}}={G}_{\mathrm{R}}^{\mathrm{itc}}\approx 0.5$), resulting in constant perceived contrast between viewing monocularly and binocularly, i.e., the summation factor = 1; (3) at the threshold level, i.e., $\widehat{T}={g}_{c}$, the summation factor $\approx 1.5$ (${G}_{\mathrm{L}}^{\mathrm{itc}}={G}_{\mathrm{R}}^{\mathrm{itc}}\approx 0.75$).

To explain monocular flank’s suppression and facilitation, we propose a model with monocular flank-to-target gain-control and gain-enhancement as shown in Fig. 5C, where flank’s gain-control and gain-enhancement depend on flank-target separations. Because the model is symmetric between the two eyes, in the following, we only describe the LE’s output. Let ${F}_{\mathrm{L}}$ be flank contrast presenting to the LE, *r* be its distance separating from the target, and ${G}_{\mathrm{L}}^{\mathrm{mfe}}$ is the LE’s gain after monocular flank enhancement. After monocular flank gain-enhancement, the LE’s output is given by, ${\widehat{T}}_{\mathrm{L}}={G}_{\mathrm{L}}^{\mathrm{mfe}}{T}_{\mathrm{L}}$ and the LE’s gain is given by,

3 $${G}_{\mathrm{L}}^{\mathrm{mfe}}=\left(1+{\left(\frac{{F}_{\mathrm{L}}}{{g}_{e}}\right)}^{\gamma }{w}_{\mathrm{mfe}}\left(r\right)\right),$$

where ${g}_{e}$ is gain-enhancement threshold (= monocular contrast detection threshold) and ${w}_{\mathrm{mfe}}\left(r\right)$ is a flank-target distance weighting function for flank’s monocular gain-enhance, given by,

4 $${w}_{\mathrm{mfe}}\left(r\right)=\frac{1}{{D}_{\mathrm{mfe}}^{{q}_{\mathrm{mfe}}}+{r}^{{q}_{\mathrm{mfe}}}}.$$

The distance weight is flattened when r < D and then decreases when r > D. Equation (4) is similar to the contrast space weighting function (CSWF) in Ding and Levi^33^ that gives the weighted contrast of a local contour when calculating the total gain-control/gain-enhancement energy.

After monocular flank’s gain-control, the LE’s output is given by,

5 $${\widehat{T}}_{\mathrm{L}}={G}_{\mathrm{L}}^{\mathrm{mfc}}{T}_{\mathrm{L}}=\frac{1}{1+{\left(\frac{{F}_{\mathrm{L}}}{{g}_{c}}\right)}^{\gamma }{w}_{\mathrm{mfc}}(r)} {T}_{\mathrm{L}},$$

where ${w}_{\mathrm{mfc}}(r)$ is a flank-target distance weighting function, given by Eq. (4) but with different parameters of ${D}_{\mathrm{mfc}}$ and ${q}_{\mathrm{mfc}}$. Balancing between flank’s monocular gain-control and gain-enhancement, the monocular flank-to-target interaction can be suppression or facilitation depending on flank-target distance. Please note that the monocular flank gain-control just described was considered a possible mechanism but turned out not to be needed in the best-fitting model (Fig. 5D). Instead interocular flank- to-target gain control and enhancement emerged as key features for a good model.

In normal vision, monocular flank’s gain-control and gain-enhancement affect monocular and binocular perception equally and have no effect on binocular summation. To explain our binocular summation data, we propose GI model: DS gain-control model with both monocular and interocular flank-target interactions (Fig. 5D). Like interocular target-target gain-control (Fig. 5A), the interocular flank-target gain-control also has two layers. In the signal layer (Black in Fig. 5D), the LE’s target signal is gain-controlled by the RE’s flank, and in the gain-control layer (Blue in Fig. 5D), the RE’s flank gain-control of LE’s target is gain-controlled by LE’s target. Similarly, interocular flank- target gain-enhancement has two layers. In the signal layer (Black in Fig. 5D), the LE’s target signal is gain-enhanced by the RE’s flank, and in the gain-enhancement layer (Red in Fig. 5D), the RE’s flank gain-enhancement of LE’s target is gain-controlled by LE’s target. The binocular output of the model shown in Fig. 5D is given by

6 $$\widehat{T}={\widehat{T}}_{\mathrm{L}}+{\widehat{T}}_{\mathrm{R}}={G}_{\mathrm{L}}^{\mathrm{ifc}}{G}_{\mathrm{L}}^{\mathrm{ife}}{G}_{\mathrm{L}}^{\mathrm{mfe}}{G}_{\mathrm{L}}^{\mathrm{itc}}{T}_{\mathrm{L}}+{G}_{\mathrm{R}}^{\mathrm{ifc}}{G}_{\mathrm{R}}^{\mathrm{ife}}{G}_{\mathrm{R}}^{\mathrm{mfe}}{G}_{\mathrm{R}}^{\mathrm{itc}}{T}_{\mathrm{R}}$$

where ${G}_{\mathrm{L}}^{\mathrm{itc}}$ and ${G}_{\mathrm{R}}^{\mathrm{itc}}$ are given by Eq. (2), and ${G}_{\mathrm{L}}^{\mathrm{mfe}}$ and ${G}_{\mathrm{R}}^{\mathrm{mfe}}$ are given by Eq. (3). The LE’s gain after interocular flank-target gain-control is given by,

7 $${G}_{\mathrm{L}}^{\mathrm{ifc}}= \frac{1}{1+\frac{{\left(\frac{{F}_{\mathrm{R}}}{{g}_{c}}\right)}^{\gamma }{w}_{\mathrm{ifc}}\left(r\right)}{1+{{\alpha }^{\gamma }\left(\frac{{T}_{\mathrm{L}}}{{g}_{c}}\right)}^{\gamma }} },$$

and the LE’s gain after interocular flank-target gain-enhancement is given by,

8 $${G}_{\mathrm{L}}^{\mathrm{ife}}=1+\frac{{\left(\frac{{F}_{\mathrm{R}}}{{g}_{e}}\right)}^{\gamma }{w}_{\mathrm{ife}}\left(r\right)}{1+{{\beta }^{\gamma }\left(\frac{{T}_{\mathrm{L}}}{{g}_{c}}\right)}^{\gamma }},$$

where $\beta$ is the relative gain-control efficiency in the gain-enhancement path (Red in Fig. 4C), and ${w}_{\mathrm{ifc}}\left(r\right)$ and ${w}_{\mathrm{ife}}\left(r\right)$ are distance weighting functions given by Eq. (4), with parameters of ${D}_{\mathrm{ifc}}$ and ${q}_{\mathrm{ifc}}$ for ${w}_{\mathrm{ifc}}\left(r\right)$, and ${D}_{\mathrm{ife}}$ and ${q}_{\mathrm{ife}}$ for ${w}_{\mathrm{ife}}\left(r\right)$. Balancing between interocular flank gain-control and gain-enhancement, the flank can exert dichoptic suppression or facilitation to the target depending on the flank-target distance. At one distance, no binocular summation might be observed because of dichoptic flank suppression, while at another distance, whole binocular summation might be observed because of dichoptic flank facilitation. Please note that, because our contrast matching data does not show monocular suppression of flanker, the monocular flank-to-target gain-control was not added to the model.

Table 3 shows the best fit of GI to contrast matching data (Fig. 4B,C). Gain-control and gain-enhancement thresholds (${g}_{c}$ and ${g}_{e}$) are in the unit of contrast threshold, and the distances ${D}_{\mathrm{mfe}}$, ${D}_{\mathrm{ifc}}$ and ${D}_{\mathrm{ife}}$ are in standard deviation units (SDU, i.e., multiples of the envelope standard deviation). Although the best fit of ${D}_{\mathrm{mfe}}$ and ${D}_{\mathrm{ifc}}$ are 0 SDU, they should be a non-zero value to avoid the singularity at *r* = 0 (this never happens in this study because the shortest distance = 1.5 SDU). The best power parameters (${q}_{\mathrm{mfe}}$, ${q}_{\mathrm{ifc}}$ and ${q}_{\mathrm{ife}}$) in distance weighting functions are between 2 and 4. They are consistent with Ding and Levi^33^, in which they found that the CSWF with *q* = 4 can give reasonable explanations of luminance binocular combination with either symmetric or asymmetric contours in the two eyes.

Pelli^39^ proposed the uncertainty model to explain the contrast-discrimination facilitation of low-contrast pedestal. His model simulations showed that the contrast discrimination threshold decreased when the pedestal increased to the detection threshold because of uncertainty reduction (UR), and then remained constant when further increasing the pedestal contrast. To fit the experimental data, we applied an analytic formula of equivalent internal noise modulated by the stimulus-induced UR, to describe the Pelli’s simulation of the uncertainty model. Let $\widehat{C}$ be binocular stimulus contrast and ${\sigma }_{0}$ be the SD of internal noise with the least uncertainty at the highest stimulus contrast. The SD of internal noise is given by:

9 $${\sigma }_{\mathrm{UR}}={\sigma }_{0}+\frac{1-{\sigma }_{0}}{1+{\left(\frac{\widehat{C}}{{C}_{0.5}}\right)}^{{p}_{\mathrm{UR}}}},$$

where ${C}_{0.5}$ is the contrast with half UR at which ${\sigma }_{\mathrm{UR}}=0.5+0.5{\sigma }_{0}$ and ${p}_{\mathrm{UR}}$ is a power parameter. Without stimulus, i.e., $\widehat{C}=0$, ${\sigma }_{\mathrm{UR}}=1$, and at high stimulus contrast, ${\sigma }_{\mathrm{UR}}\approx {\sigma }_{0}$. To explain the pedestal/flank masking effect, we proposed stimulus-induce MN. The SD of MN is given by:

10 $${\sigma }_{\mathrm{MN}}=b(\widehat{C}{)}^{{p}_{\mathrm{MN}}}.$$

Let *R*2 and *R*1 be two contrast responses of a 2AFC task for contrast detection/discrimination with and without targets, respectively, and ${\sigma }_{\mathrm{UR}2}$ and ${\sigma }_{\mathrm{MN}2}$ be internal noise for *R*2 response, and ${\sigma }_{\mathrm{UR}1}$ and ${\sigma }_{\mathrm{MN}1}$ be internal noise for *R*1 response. The contrast detection/discrimination thresholds are the solution of the following equation:

11 $${R}_{2}-{R}_{1}=\sqrt{{\sigma }_{\mathrm{UR}1}^{2}+{\sigma }_{\mathrm{MN}1}^{2}+{\sigma }_{\mathrm{UR}2}^{2}+{\sigma }_{\mathrm{MN}2}^{2}}.$$

The details of model predictions of contrast detection/discrimination are given in Appendix C.

## Supplementary Information

Supplementary Information.

## References

[1] Blake R, Fox R (1973). The psychophysical inquiry into binocular SUlnmation. Percept. Psychophys..

[2] Blake R, Sloane M, Fox R (1981). Further developments in binocular summation. Percept. Psychophys..

[3] Baker DH, Lygo FA, Meese TS, Georgeson MA (2018). Binocular summation revisited: Beyond radical2. Psychol. Bull..

[4] Ding J, Sperling G (2006). A gain-control theory of binocular combination. Proc. Natl. Acad. Sci. U. S. A..

[5] Baker DH, Wallis SA, Georgeson MA, Meese TS (2012). Nonlinearities in the binocular combination of luminance and contrast. Vis. Res..

[6] Meese TS, Georgeson MA, Baker DH (2006). Binocular contrast vision at and above threshold. J. Vis..

[7] Blake R, Wilson H (2011). Binocular vision. Vis. Res..

[8] Polat U, Sagi D (1994). Spatial interactions in human vision: From near to far via experience-dependent cascades of connections. Proc. Natl. Acad. Sci. U. S. A..

[9] Polat U, Sagi D (1994). The architecture of perceptual spatial interactions. Vis. Res..

[10] Lev M, Polat U (2011). Collinear facilitation and suppression at the periphery. Vis. Res..

[11] Huang PC, Hess RF, Dakin SC (2006). Flank facilitation and contour integration: Different sites. Vis. Res..

[12] Maehara G, Huang PC, Hess RF (2010). The effects of flankers on contrast detection and discrimination in binocular, monocular, and dichoptic presentations. J. Vis..

[13] Levi DM (2008). Crowding—An essential bottleneck for object recognition: A mini-review. Vis. Res..

[14] Petrov Y, Carandini M, McKee S (2005). Two distinct mechanisms of suppression in human vision. J. Neurosci..

[15] Levi DM, Carney T (2011). The effect of flankers on three tasks in central, peripheral, and amblyopic vision. J. Vis..

[16] Levi DM, Klein SA, Hariharan S (2002). Suppressive and facilitatory spatial interactions in foveal vision: Foveal crowding is simple contrast masking. J. Vis..

[17] Hess RF, Dakin SC (1997). Absence of contour linking in peripheral vision. Nature.

[18] Lev M, Polat U (2015). Space and time in masking and crowding. J. Vis..

[19] Legge GE, Foley JM (1980). Contrast masking in human vision. J. Opt. Soc. Am..

[20] Maehara G, Goryo K (2005). Binocular, monocular and dichoptic pattern masking. Opt. Rev..

[21] Levi DM, Harwerth RS, Smith EL (1979). Humans deprived of normal binocular vision have binocular interactions tuned to size and orientation. Science.

[22] Legge GE (1979). Spatial frequency masking in human vision: Binocular interactions. J. Opt. Soc. Am..

[23] Legge GE (1984). Binocular contrast summation—I. Detection and discrimination. Vis. Res..

[24] Chen CC, Tyler CW (2008). Excitatory and inhibitory interaction fields of flankers revealed by contrast-masking functions. J. Vis..

[25] Meese TS, Challinor KL, Summers RJ (2008). A common contrast pooling rule for suppression within and between the eyes. Vis. Neurosci..

[26] Foley JM (1994). Human luminance pattern-vision mechanisms: Masking experiments require a new model. J. Opt. Soc. Am. A Opt. Image Sci. Vis..

[27] Holmes DJ, Meese TS (2004). Grating and plaid masks indicate linear summation in a contrast gain pool. J. Vis..

[28] Ding J, Klein SA, Levi DM (2013). Binocular combination of phase and contrast explained by a gain-control and gain-enhancement model. J. Vis..

[29] Legge GE (1984). Binocular contrast summation—II. Quadratic summation. Vis. Res..

[30] Legge GE, Rubin GS (1981). Binocular interactions in suprathreshold contrast perception. Percept. Psychophys..

[31] Ding J, Klein SA, Levi DM (2013). Binocular combination in abnormal binocular vision. J. Vis..

[32] Ding J, Levi DM (2016). Binocular contrast discrimination needs monocular multiplicative noise. J. Vis..

[33] Ding J, Levi DM (2017). Binocular combination of luminance profiles. J. Vis..

[34] Ding, J. & Sperling, G. In *Computational Vision in Neural and Machine Systems* (eds Harris & Jenkin, M.) 257–305 (Cambridge Unversity Press, 2007).

[35] Yehezkel O, Ding J, Sterkin A, Polat U, Levi DM (2016). Binocular combination of stimulus orientation. R. Soc. Open Sci..

[36] Hou F, Huang CB, Liang J, Zhou Y, Lu ZL (2013). Contrast gain-control in stereo depth and cyclopean contrast perception. J. Vis..

[37] Ding J, Levi DM (2021). A unified model for binocular fusion and depth perception. Vis. Res..

[38] Chen PY, Chen CC, Tyler CW (2021). A gain-control disparity energy model for perceived depth from disparity. Vis. Res..

[39] Pelli DG (1985). Uncertainty explains many aspects of visual contrast detection and discrimination. J. Opt. Soc. Am. A.

[40] McIlhagga W (2004). Denoising and contrast constancy. Vis. Res..

[41] Petrov Y, Verghese P, McKee SP (2006). Collinear facilitation is largely uncertainty reduction. J. Vis..

[42] Tyler CW, Chen CC (2000). Signal detection theory in the 2AFC paradigm: Attention, channel uncertainty and probability summation. Vis. Res..

[43] Meese TS, Challinor KL, Summers RJ, Baker DH (2009). Suppression pathways saturate with contrast for parallel surrounds but not for superimposed cross-oriented masks. Vis. Res..

[44] Meese TS, Summers RJ (2012). Theory and data for area summation of contrast with and without uncertainty: Evidence for a noisy energy model. J. Vis..

[45] Meese TS, Holmes DJ, Challinor KL (2007). Remote facilitation in the Fourier domain. Vis. Res..

[46] Meese TS, Baker DH (2009). Cross-orientation masking is speed invariant between ocular pathways but speed dependent within them. J. Vis..

[47] Chen CC, Tyler CW (2002). Lateral modulation of contrast discrimination: Flanker orientation effects. J Vis.

[48] Yu C, Klein SA, Levi DM (2002). Facilitation of contrast detection by cross-oriented surround stimuli and its psychophysical mechanisms. J. Vis..

[49] Akaike, H. In *Automatic Control, IEEE Transactions.* 716–723 (IEEE).

[50] Levi DM, Hariharan S, Klein SA (2002). Suppressive and facilitatory spatial interactions in peripheral vision: Peripheral crowding is neither size invariant nor simple contrast masking. J. Vis..

[51] Siman-Tov Z, Lev M, Polat U (2021). Binocular summation is affected by crowding and tagging. Sci. Rep..

[52] Levi DM, Carney T (2009). Crowding in peripheral vision: Why bigger is better. Curr. Biol..

[53] Whitney D, Levi DM (2011). Visual crowding: A fundamental limit on conscious perception and object recognition. Trends Cogn. Sci..

[54] Enroth-Cugell C, Shapley RM (1973). Adaptation and dynamics of cat retinal ganglion cells. J. Physiol..

[55] Shapley R, Enroth-Cugell C, Bonds AB, Kirby A (1972). Gain control in the retina and retinal dynamics. Nature.

[56] Shapley R, Victor JD (1979). The contrast gain control of the cat retina. Vis. Res..

[57] Carandini M, Heeger DJ, Movshon JA (1997). Linearity and normalization in simple cells of the macaque primary visual cortex. J. Neurosci..

[58] Levi L (1969). Automatic gain control model for vision. Nature.

[59] Ohzawa I, Sclar G, Freeman RD (1985). Contrast gain control in the cat's visual system. J. Neurophysiol..

[60] Sclar G, Ohzawa I, Freeman RD (1985). Contrast gain control in the kitten's visual system. J. Neurophysiol..

[61] Astle AT, Li RW, Webb BS, Levi DM, McGraw PV (2013). A Weber-like law for perceptual learning. Sci. Rep..

[62] Baker DH, Meese TS, Georgeson MA (2007). Binocular interaction: Contrast matching and contrast discrimination are predicted by the same model. Spat. Vis..

[63] Sit YF, Chen Y, Geisler WS, Miikkulainen R, Seidemann E (2009). Complex dynamics of V1 population responses explained by a simple gain-control model. Neuron.

[64] Tso DY, Miller RA (2019). Homeostatic control of interocular balance revealed with contrast mismatch. J. Vis..

[65] Polat U, Mizobe K, Pettet MW, Kasamatsu T, Norcia AM (1998). Collinear stimuli regulate visual responses depending on cell's contrast threshold. Nature.

[66] Malach R, Amir Y, Harel M, Grinvald A (1993). Relationship between intrinsic connections and functional architecture revealed by optical imaging and in vivo targeted biocytin injections in primate striate cortex. Proc. Natl. Acad. Sci. U. S. A..

[67] Ohzawa, I., & Freeman, R.D. Monocular and binocular mechanisms of contrast gain control. In Computational Vision Based on Neurobiology. S.P.I.E. Proceedings, Vol. 2054 (1994).

[68] Truchard AM, Ohzawa I, Freeman RD (2000). Contrast gain control in the visual cortex: Monocular versus binocular mechanisms. J. Neurosci..

[69] Smith EL, Chino Y, Ni J, Cheng H (1997). Binocular combination of contrast signals by striate cortical neurons in the monkey. J. Neurophysiol..

[70] Longordo F, To MS, Ikeda K, Stuart GJ (2013). Sublinear integration underlies binocular processing in primary visual cortex. Nat. Neurosci..

