# Supplementary material for: Nearby contours abolish the binocular advantage
Source: Sci Rep. 2021 Aug 19;11:16920. doi: 10.1038/s41598-021-96053-9 (PMC8376993; doi:10.1038/s41598-021-96053-9)
Supplement: Supplementary file 1 — Supplementary Information. [file 41598_2021_96053_MOESM1_ESM.pdf]

Nearby contours abolish binocular abolish the binocular advantage.

Maria Lev[1,2,3], Jian Ding[2,3], Uri Polat[1,3] & Dennis M. Levi[2,3*]

Supplementary Information

A. Alternative models for fitting contrast matching data

We tested multiple models with different combinations of flank-target interactions and found GI (Figure 5D) is the best one. In the following, we described all these alternative models (AM). Let T_L and T_R be targets' and F_L and F_R be flanks' contrasts presenting to the two eyes.

AM 1. DS gain-control model [1,2] (Figure 5A) without flank-to-target interactions. The model binocular output is given by:

$$\hat{T} = G_L^{itc} T_L + G_R^{itc} T_R \quad . \qquad (A1)$$

G_L^{itc} and G_R^{itc} are two eyes' gains after interocular target-target gain-controls, given by,

$$G_L^{itc} = \frac{1}{1+\frac{\left(\frac{T_R}{g_C}\right)^\gamma}{1+\alpha^\gamma\left(\frac{T_L}{g_C}\right)^\gamma}} \text{ and } G_R^{itc} = \frac{1}{1+\frac{\left(\frac{T_L}{g_C}\right)^\gamma}{1+\alpha^\gamma\left(\frac{T_R}{g_C}\right)^\gamma}} \qquad (A2)$$

AM 2. DS gain-control model with inputs of both targets and flankers that perform as equivalent weak pedestals (wPed model: Fig. A1 A). The model binocular output is given by:

$$G_L^{itc} = \frac{1}{1+\frac{\left(\frac{T_R+w_F F_R}{g_C}\right)^\gamma}{1+\alpha^\gamma\left(\frac{T_L+w_F F_L}{g_C}\right)^\gamma}} \text{ and } G_R^{itc} = \frac{1}{1+\frac{\left(\frac{T_L+w_F F_L}{g_C}\right)^\gamma}{1+\alpha^\gamma\left(\frac{T_R+w_F F_R}{g_C}\right)^\gamma}} \qquad (A3)$$

where F_L and F_R are flank contrast presenting to the LE and RE respectively, and w_F is a flank-target distance weighting function to transfer a flanker to an equivalent weak pedestal. Let *r* be the distance in SDU, w_F is given by,

$$w_F(r) = \frac{1}{1+r^{q_F}} \quad . \qquad (A4)$$

When $r = 0$, $w_F(0) = 1$, i.e., the flanker becomes a pedestal.

AM 3. DS model with monocular flank-to-target gain-control (Figure A1 B). With monocular flanks' gain-control, the monocular outputs of the DS model are given by:

$$\hat{T} = G_L^{mfc} G_L^{itc} T_L + G_R^{mfc} G_R^{itc} T_R \quad . \qquad (A5)$$

where G_L^{itc} and G_R^{itc} are given by Eq. A2. G_L^{mfc} and G_R^{mfc} are two eyes' gains after monocular flank's gain-control, given by,

$$G_L^{mfc} = \frac{1}{1+\left(\frac{F_L}{g_C}\right)^\gamma w_{mfc}(r)} \text{ and } G_R^{mfc} = \frac{1}{1+\left(\frac{F_R}{g_C}\right)^\gamma w_{mfc}(r)} \qquad (A6)$$

where $w_{mfc}(r)$ is a flank-target distance weighting function for monocular flank's gain-control, given by,

$$w_{mfc}(r) = \frac{1}{D_{mfc}^{q_{mfc}}+r^{q_{mfc}}} \quad . \qquad (A7)$$

AM 4. DS model with monocular flank gain-enhancement (Figure A1 C).

$$\hat{T} = G_L^{\text{mfe}} G_L^{\text{itc}} T_L + G_R^{\text{mfe}} G_R^{\text{itc}} T_R \quad . \qquad \text{(A8)}$$

where G_L^{itc} and G_R^{itc} are given by Eq. A2. G_L^{mfe} and G_R^{mfe} are two eyes' gains after monocular flank's gain-enhancement, given by,

$$G_L^{\text{mfe}} = 1 + \left(\frac{F_L}{g_c}\right)^{\gamma} w_{\text{mfe}}(r) \quad \text{and} \quad G_R^{\text{mfe}} = 1 + \left(\frac{F_R}{g_c}\right)^{\gamma} w_{\text{mfe}}(r) \quad , \qquad \text{(A9)}$$

where $w_{\text{mfe}}(r)$ is a flank-target distance weighting function for flank's monocular gain-enhancement, given by,

$$w_{\text{mfe}}(r) = \frac{1}{D_{\text{mfe}}^{q_{\text{mfe}}} + r^{q_{\text{mfe}}}} \quad . \qquad \text{(A10)}$$

AM 5. DS model with monocular flank's gain-control and gain-enhancement (Figure A1 D).

$$\hat{T} = G_L^{\text{mfc}} G_L^{\text{mfe}} G_L^{\text{itc}} T_L + G_R^{\text{mfc}} G_R^{\text{mfe}} G_R^{\text{itc}} T_R \quad . \qquad \text{(A11)}$$

where G_L^{itc} and G_R^{itc} are given by Eq. A2, G_L^{mfc} and G_R^{mfc} are given by Eq. A6, and G_L^{mfe} and G_R^{mfe} are given by Eq. A9.

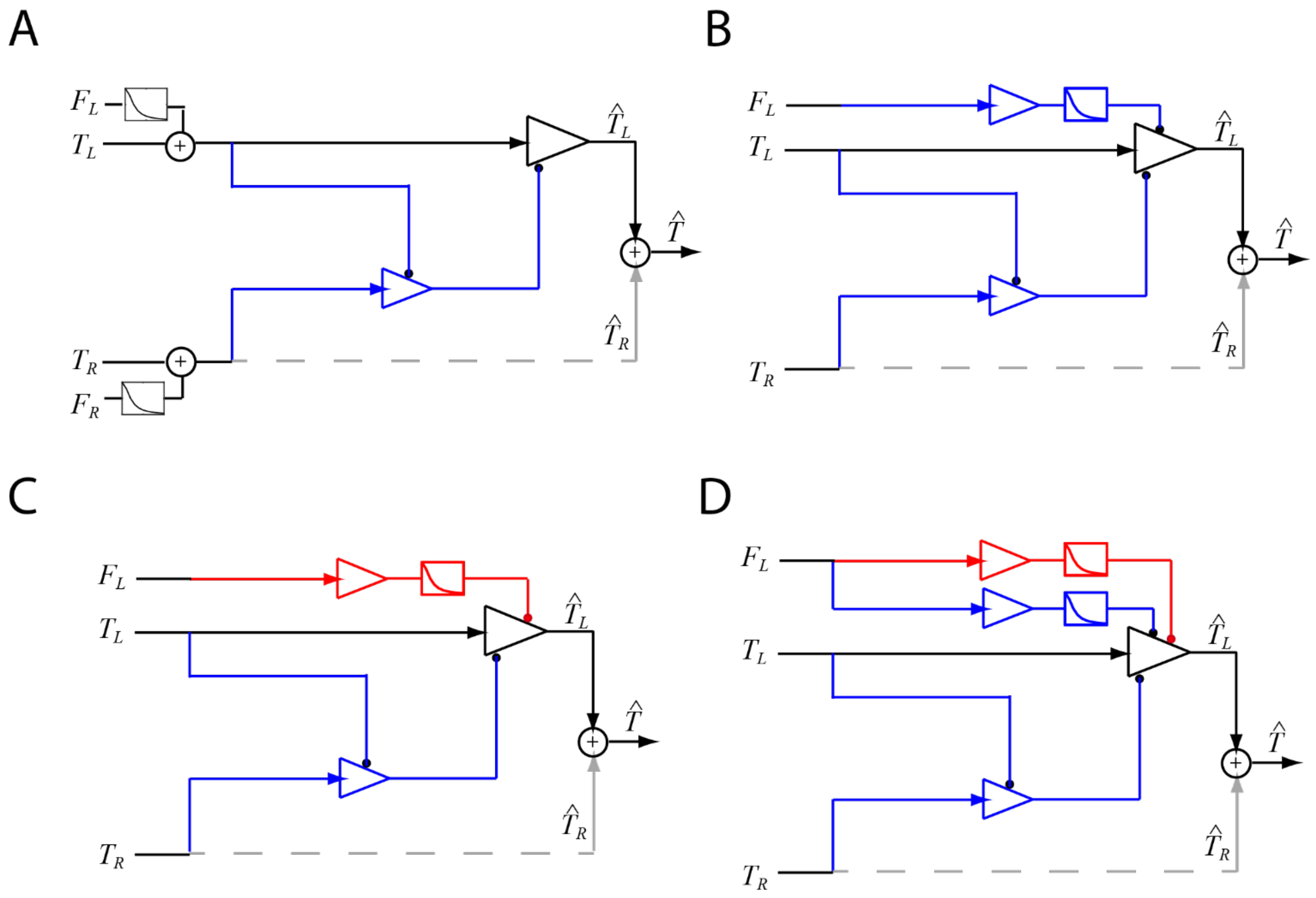

Figure A1. DS gain-control model [4,35] with (A) a flanker as an equivalent weak pedestal (AM2); (B) monocular flank-to-target gain-control (AM3); (C) monocular flank-to-target gain-enhancement (AM4); (D) both monocular flank-to-target gain-control and gain-enhancement (AM5).

A

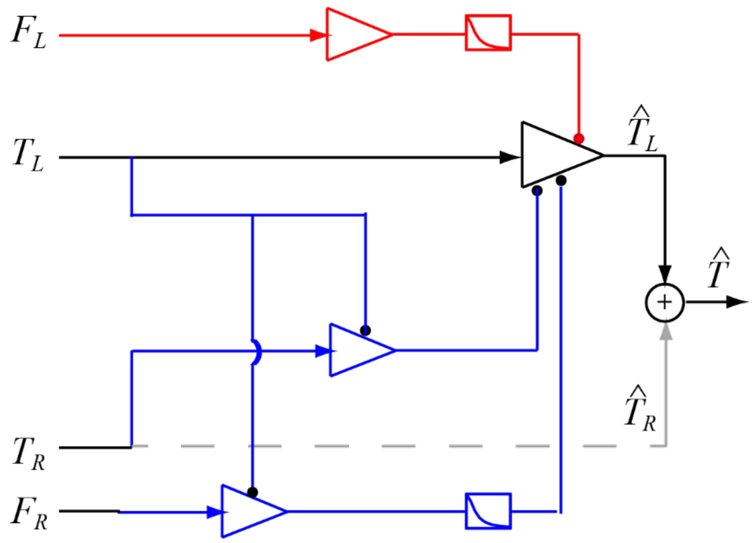

B

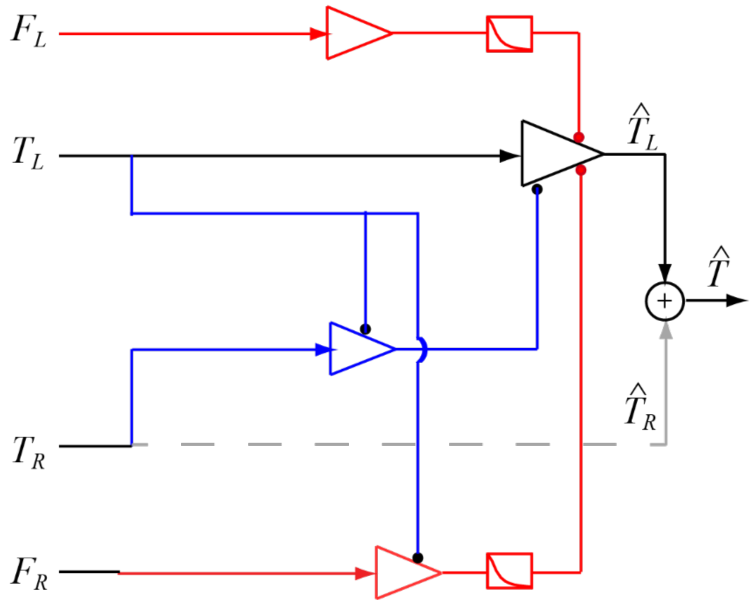

Figure A2. (A) AM 6 (B) AM 7.

AM 6. DS model plus (1) monocular flank's enhancement; (2) interocular flank's gain-control with target's gain-control of flank's gain-control. (Figure A2 A).

$$\hat{T} = G_L^{\text{ifc}} G_L^{\text{mfe}} G_L^{\text{itc}} T_L + G_R^{\text{ifc}} G_R^{\text{mfe}} G_R^{\text{itc}} T_R \quad . \qquad \text{(A12)}$$

where G_L^{itc} and G_R^{itc} are given by Eq. A2, and G_L^{mfe} and G_R^{mfe} are given by Eq. A9. G_L^{ifc} and G_R^{ifc} are two eyes' gains after interocular flank's gain-control and target's gain-control of flank's gain-control, given by,

$$G_L^{\text{ifc}} = \frac{1}{1+\frac{\left(\frac{F_R}{gc}\right)^{\gamma} w_{\text{ifc}}(r)}{1+\alpha^{\gamma}\left(\frac{T_L}{ac}\right)^{\gamma}}} \quad \text{and} \quad G_R^{\text{ifc}} = \frac{1}{1+\frac{\left(\frac{F_L}{gc}\right)^{\gamma} w_{\text{ifc}}(r)}{1+\alpha^{\gamma}\left(\frac{T_R}{ac}\right)^{\gamma}}} \quad . \qquad \text{(A13)}$$

where $w_{\text{ifc}}(r)$ is a flank-target distance weighting function for interocular flank's gain-control, given by,

$$w_{\text{ifc}}(r) = \frac{1}{D_{\text{ifc}}^{q_{\text{ifc}}} + r^{q_{\text{ifc}}}} \quad . \qquad \text{(A14)}$$

AM 7. DS model plus (1) monocular flank's enhancement; (2) interocular flank's gain-enhancement with target's gain-control of flank's gain-enhancement (Figure A2 B).

$$\hat{T} = G_L^{\text{ife}} G_L^{\text{mfe}} G_L^{\text{itc}} T_L + G_R^{\text{ife}} G_R^{\text{mfe}} G_R^{\text{itc}} T_R \quad . \qquad \text{(A15)}$$

where G_L^{itc} and G_R^{itc} are given by Eq. A2, and G_L^{mfe} and G_R^{mfe} are given by Eq. A9. G_L^{ife} and G_R^{ife} are two eyes' gains after interocular flank's gain-enhancement and target's gain-control of flank's gain-enhancement, given by,

$$G_L^{\text{ife}} = 1 + \frac{\left(\frac{F_R}{ge}\right)^{\gamma} w_{\text{ife}}(r)}{1+\beta^{\gamma}\left(\frac{T_L}{ac}\right)^{\gamma}} \quad \text{and} \quad G_R^{\text{ife}} = 1 + \frac{\left(\frac{F_L}{ge}\right)^{\gamma} w_{\text{ife}}(r)}{1+\beta^{\gamma}\left(\frac{T_R}{ac}\right)^{\gamma}} \quad . \qquad \text{(A16)}$$

where $w_{\text{ife}}(r)$ is a flank-target distance weighting function for interocular flank's gain-enhancement, given by,

$$w_{\text{ife}}(r) = \frac{1}{D_{\text{ife}}^{q_{\text{ife}}} + r^{q_{\text{ife}}}} \quad . \qquad \text{(A17)}$$

AM 8. DS model plus (1) monocular flank's enhancement; (2) interocular flank's gain-enhancement without target's gain-control of flank's gain-enhancement; (3) interocular flank's gain-control without target's gain-control of flank's gain-control (Figure A3 A).

$$\hat{T} = G_L^{\text{ifc}} G_L^{\text{ife}} G_L^{\text{mfe}} G_L^{\text{itc}} T_L + G_R^{\text{ifc}} G_R^{\text{ife}} G_R^{\text{mfe}} G_R^{\text{itc}} T_R \quad , \qquad \text{(A18)}$$

where G_L^{itc} and G_R^{itc} are given by Eq. A2, G_L^{mfe} and G_R^{mfe} are given by Eq. A9, and

$$G_L^{\text{ife}} = 1 + \left(\frac{F_R}{g_e}\right)^{\gamma} w_{\text{ife}}(r) \quad \text{and} \quad G_R^{\text{ife}} = 1 + \left(\frac{F_L}{g_e}\right)^{\gamma} w_{\text{ife}}(r) \qquad \text{(A19)}$$

$$G_L^{\text{ifc}} = \frac{1}{1+\left(\frac{F_R}{ac}\right)^{\gamma} w_{\text{ifc}}(r)} \quad \text{and} \quad G_R^{\text{ifc}} = \frac{1}{1+\left(\frac{F_L}{ac}\right)^{\gamma} w_{\text{ifc}}(r)} \quad . \qquad \text{(A20)}$$

AM 9. DS model plus (1) monocular flank's enhancement; (2) interocular flank's gain-enhancement without target's gain-control of flank's gain-enhancement; (3) interocular flank's gain-control with target's gain-control of flank's gain-control (Figure A3 B). The model output is given by Eq. A18, where G_L^{itc} and G_R^{itc} are given by Eq. A2, G_L^{mfe} and G_R^{mfe} are given by Eq. A9, G_L^{ife} and G_R^{ife} are given by Eq. A19, and G_L^{ifc} and G_R^{ifc} are given by Eq. A13.

AM 10. DS model plus (1) monocular flank's enhancement; (2) interocular flank's gain-enhancement with target's gain-control of flank's gain-enhancement; (3) interocular flank's gain-control without target's gain-control of flank's gain-control (Figure A3 C). The model output is

given by Eq. A18, where G_L^{itc} and G_R^{itc} are given by Eq. A2, G_L^{mfe} and G_R^{mfe} are given by Eq. A9, G_L^{ife} and G_R^{ife} are given by Eq. A16, and G_L^{ifc} and G_R^{ifc} are given by Eq. A20.

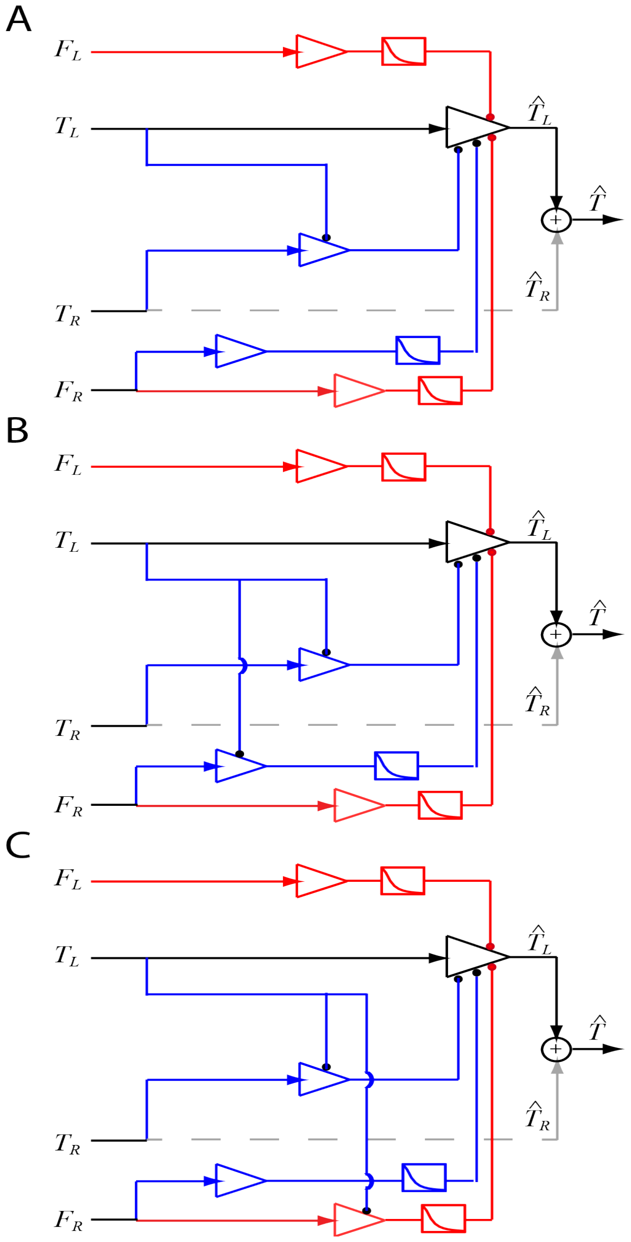

Figure A3. (A) AM 8, (B) AM 9, and (C) AM 10.

AM 11. The gain-control model with flanker-target interactions: DS model plus (1) monocular flank's enhancement; (2) flank's gain-enhancement of binocularly combined target with interocular target's gain-control of flank's gain-enhancement; (3) interocular flank's gain-control with target's gain-control of flank's gain-control (Figure A4. A). The model output is given by:

$\hat{T} = G_L^{ife}\, G_R^{ife}\left(G_L^{ifc} G_L^{mfe} G_L^{itc} T_L + G_R^{ifc} G_R^{mfe} G_R^{itc} T_R\right)$, (A21)

where $G_{\mathrm{L}}^{\mathrm{itc}}$ and $G_{\mathrm{R}}^{\mathrm{itc}}$ are given by Eq. A2, $G_{\mathrm{L}}^{\mathrm{mfe}}$ and $G_{\mathrm{R}}^{\mathrm{mfe}}$ are given by Eq. A9, $G_{\mathrm{L}}^{\mathrm{ife}}$ and $G_{\mathrm{R}}^{\mathrm{ife}}$ are given by Eq. A16, and $G_{\mathrm{L}}^{\mathrm{ifc}}$ and $G_{\mathrm{R}}^{\mathrm{ifc}}$ are given by Eq. A13.

AM 12. DS model plus (1) monocular flank's enhancement; (2) flank's gain-enhancement of binocularly combined target with interocular target's gain-control of flank's gain-enhancement; (3) flank's gain-control of binocularly combined target with interocular target's gain-control of flank's gain-control (Figure A4. B). The model output is given by:

$$\hat{T} = G_{\mathrm{L}}^{\mathrm{ifc}} G_{\mathrm{L}}^{\mathrm{ife}} G_{\mathrm{R}}^{\mathrm{ifc}} G_{\mathrm{R}}^{\mathrm{ife}} \left(G_{\mathrm{L}}^{\mathrm{mfe}} G_{\mathrm{L}}^{\mathrm{itc}} T_{\mathrm{L}} + G_{\mathrm{R}}^{\mathrm{mfe}} G_{\mathrm{R}}^{\mathrm{itc}} T_{\mathrm{R}}\right), \quad \text{(A22)}$$

where $G_{\mathrm{L}}^{\mathrm{itc}}$ and $G_{\mathrm{R}}^{\mathrm{itc}}$ are given by Eq. A2, $G_{\mathrm{L}}^{\mathrm{mfe}}$ and $G_{\mathrm{R}}^{\mathrm{mfe}}$ are given by Eq. A9, $G_{\mathrm{L}}^{\mathrm{ife}}$ and $G_{\mathrm{R}}^{\mathrm{ife}}$ are given by Eq. A16, and $G_{\mathrm{L}}^{\mathrm{ifc}}$ and $G_{\mathrm{R}}^{\mathrm{ifc}}$ are given by Eq. A13.

AM 13 (aGI). Alternative GI model: DS model plus (1) monocular flank's enhancement; (2) flank's gain-enhancement of binocularly combined target with monocular target's gain-control of flank's gain-enhancement; (3) flank's gain-control of binocularly combined target with monocular target's gain-control of flank's gain-control (Figure A4. C). The model output is given by Eq. A21, where $G_{\mathrm{L}}^{\mathrm{itc}}$ and $G_{\mathrm{R}}^{\mathrm{itc}}$ are given by Eq. A2, $G_{\mathrm{L}}^{\mathrm{mfe}}$ and $G_{\mathrm{R}}^{\mathrm{mfe}}$ are given by Eq. A9, $G_{\mathrm{L}}^{\mathrm{ife}}$ and $G_{\mathrm{R}}^{\mathrm{ife}}$ are given by

$$G_{\mathrm{L}}^{\mathrm{ife}} = 1 + \frac{\left(\frac{F_{\mathrm{R}}}{ge}\right)^{\gamma} w_{\mathrm{ife}}(r)}{1+\beta^{\gamma}\left(\frac{T_{\mathrm{R}}}{ac}\right)^{\gamma}} \quad \text{and} \quad G_{\mathrm{R}}^{\mathrm{ife}} = 1 + \frac{\left(\frac{F_{\mathrm{L}}}{ge}\right)^{\gamma} w_{\mathrm{ife}}(r)}{1+\beta^{\gamma}\left(\frac{T_{\mathrm{L}}}{ac}\right)^{\gamma}}, \quad \text{(A23)}$$

and $G_{\mathrm{L}}^{\mathrm{ifc}}$ and $G_{\mathrm{R}}^{\mathrm{ifc}}$ are given by

$$G_{\mathrm{L}}^{\mathrm{ifc}} = \frac{1}{1+\frac{\left(\frac{F_{\mathrm{R}}}{gc}\right)^{\gamma} w_{\mathrm{ifc}}(r)}{1+\alpha^{\gamma}\left(\frac{T_{\mathrm{R}}}{ac}\right)^{\gamma}}} \quad \text{and} \quad G_{\mathrm{R}}^{\mathrm{ifc}} = \frac{1}{1+\frac{\left(\frac{F_{\mathrm{L}}}{gc}\right)^{\gamma} w_{\mathrm{ifc}}(r)}{1+\alpha^{\gamma}\left(\frac{T_{\mathrm{L}}}{ac}\right)^{\gamma}}}. \quad \text{(A24)}$$

AM 14 (GI). The gain-control model with flanker-target interactions: DS model plus (1) monocular flank's enhancement; (2) interocular flank's gain-enhancement with target's gain-control of flank's gain-enhancement; (3) interocular flank's gain-control with target's gain-control of flank's gain-control (Figure 5C). The model output is given by Eq. A18, where $G_{\mathrm{L}}^{\mathrm{itc}}$ and $G_{\mathrm{R}}^{\mathrm{itc}}$ are given by Eq. A2, $G_{\mathrm{L}}^{\mathrm{mfe}}$ and $G_{\mathrm{R}}^{\mathrm{mfe}}$ are given by Eq. A9, $G_{\mathrm{L}}^{\mathrm{ife}}$ and $G_{\mathrm{R}}^{\mathrm{ife}}$ are given by Eq. A16, and $G_{\mathrm{L}}^{\mathrm{ifc}}$ and $G_{\mathrm{R}}^{\mathrm{ifc}}$ are given by Eq. A13.

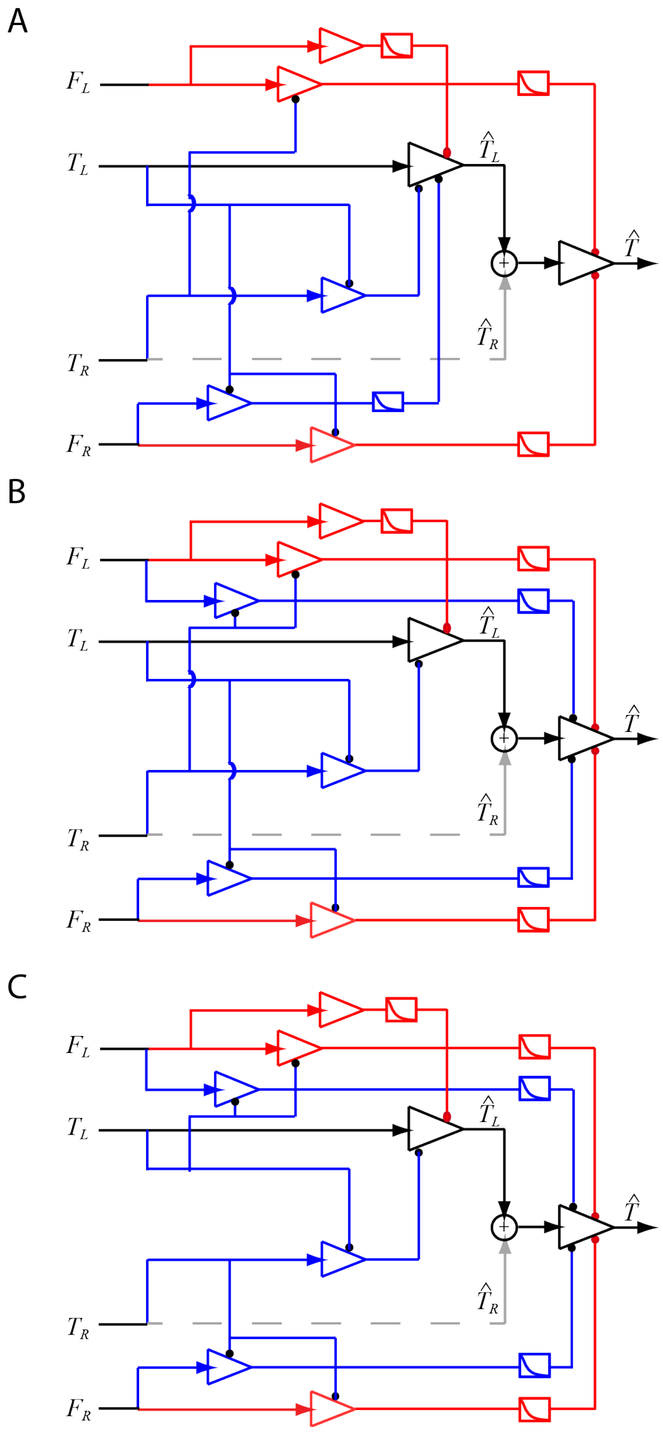

Figure A4. (A) AM 11. (B) AM12. (C) AM13 (aGI).

Model comparison. Table A1 shows fitting statistics of model AMs 1-13. The best fitting model is AM 13 (GI model). To better compare GI (AM 13), aGI (AM 12) and AM 11 models, Figs. A5 AB shows AM11 and aGI under monocular condition, and Figs. CD shows GI and aGI under dichoptic condition.

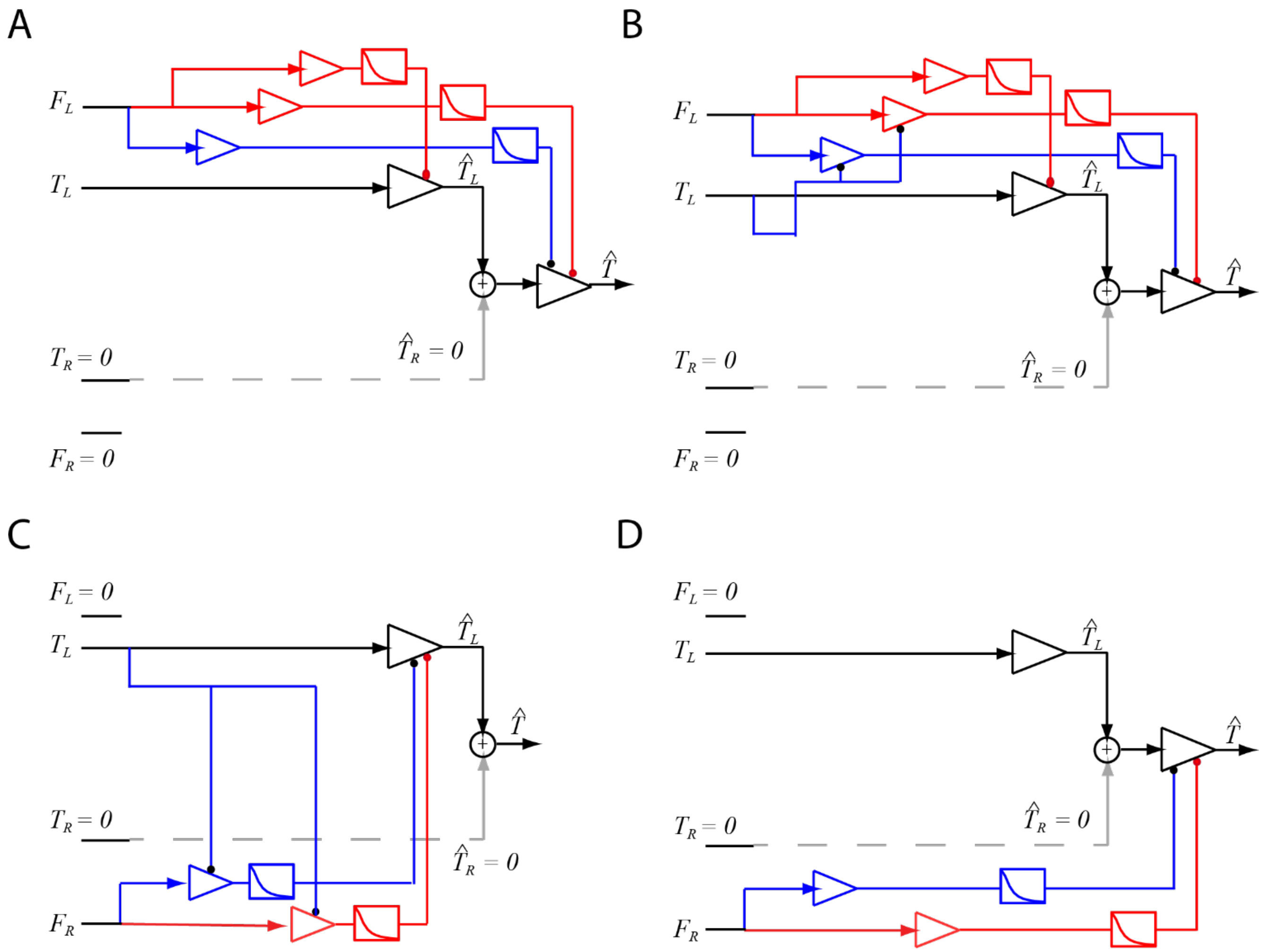

Figure A5. Comparison of GI, aGI and AM 11 models. (A) AM 11 under monocular condition, e.g., both target and flanker are presented only to the left eye ($T_R = 0$ and $F_R = 0$). (B) aGI model (AM 12) under monocular condition. (C) GI model (AM 13) under dichoptic condition -- target and flanker are presented to the different eyes (e.g., $T_R = 0$ and $F_L = 0$). (D) aGI model under dichoptic condition.

Supplementary Information B. The AIC for comparison of different models

We used the Akaike Information Criterion (AIC)[3], a measure of the relative goodness of fit of a statistical model developed by Akaike[4], to compare different models. Let K be the number of estimated parameters in the model and L_{Max} be the maximized value of the likelihood function for the model, AIC is defined as $AIC = 2K - 2\ln L_{Max}$. Assuming that the errors are normally distributed and independent, after ignoring the constant term, AIC is given by

$$AIC = N\ln\left(\frac{\chi^2}{N}\right) + 2K, \qquad \text{(B1)}$$

where χ^2 is the residual sum of square in the least squares fitting and N is the number of observed data points. To give a greater penalty for additional parameters, we applied a correction for finite sample sizes (AICc) [71]which is given by,

$$\mathrm{AICc} = \mathrm{AIC} + \frac{2\mathrm{K}(\mathrm{K}+1)}{\mathrm{N}-\mathrm{K}-1}. \qquad \text{(B2)}$$

For the set of *R* models, the one with the lowest AICc score is most likely to be the best model of those considered. The relative likelihood of model *i* is proportional to $\exp(-0.5\Delta_i)$, where Δ_i is the AICc difference between model *i* and the best model (with the lowest AICc). Given the data and the set of *R* models, the relative likelihood or Akaike weight [71], given by:

$$w_i = \frac{\exp(-0.5\Delta_i)}{\sum_{r=1}^{R}\exp(-0.5\Delta_r)} \quad . \qquad \text{(B3)}$$

Supplementary Information C. Model and Modeling for contrast detection and discrimination

GUM model for contrast discrimination

Let $\hat{C}_2$ and $\hat{C}_1$ be binocular contrast output of the DS model with and without targets, respectively. Based on Eqs. A1 and A2, we have:

$$\hat{C}_1 = \frac{1}{1+\frac{\left(\frac{Ped_\mathrm{R}}{gc}\right)^\gamma}{1+\alpha^\gamma\left(\frac{Ped_\mathrm{L}}{gc}\right)^\gamma}} Ped_\mathrm{L} + \frac{1}{1+\frac{\left(\frac{Ped_\mathrm{L}}{gc}\right)^\gamma}{1+\alpha^\gamma\left(\frac{Ped_\mathrm{R}}{gc}\right)^\gamma}} Ped_\mathrm{R} \qquad \text{(C1)}$$

and

$$\hat{C}_2 = \frac{1}{1+\frac{\left(\frac{T_\mathrm{R}+Ped_\mathrm{R}}{gc}\right)^\gamma}{1+\alpha^\gamma\left(\frac{T_\mathrm{L}+Ped_\mathrm{L}}{gc}\right)^\gamma}} (T_\mathrm{L}+Ped_\mathrm{L}) + \frac{1}{1+\frac{\left(\frac{T_\mathrm{L}+Ped_\mathrm{L}}{gc}\right)^\gamma}{1+\alpha^\gamma\left(\frac{T_\mathrm{R}+Ped_\mathrm{R}}{gc}\right)^\gamma}} (T_\mathrm{R}+Ped_\mathrm{R}) \quad , \qquad \text{(C2)}$$

where Ped_L and Ped_R are pedestal contrast presented to the LE and RE, respectively. Based on Eqs. 9 and 10, the internal noises are given by:

$$\sigma_\mathrm{UR1} = \sigma_0 + \frac{1-\sigma_0}{1+\left(\frac{\hat{C}_1}{C_{0.5}}\right)^{p_\mathrm{UR}}} \quad , \qquad \text{(C3)}$$

$$\sigma_\mathrm{MN1} = b(\hat{C}_1)^{p_\mathrm{MN}} \quad . \qquad \text{(C4)}$$

$$\sigma_\mathrm{UR2} = \sigma_0 + \frac{1-\sigma_0}{1+\left(\frac{\hat{C}_2}{C_{0.5}}\right)^{p_\mathrm{UR}}} \quad , \qquad \text{(C5)}$$

$$\sigma_\mathrm{MN2} = b(\hat{C}_2)^{p_\mathrm{MN}} \quad . \qquad \text{(C6)}$$

where $\hat{C}_1$ and $\hat{C}_2$ are given by Eqs. C1 and C2. For monocular target only condition, e.g., $Ped_\mathrm{L} = Ped_\mathrm{R} = 0$, $T_\mathrm{L} = 0$, and $T_\mathrm{R} = T$, we have $\sigma_\mathrm{UR1,T} = 1$, $\sigma_\mathrm{MN1,T} = 0$, and

$$\sigma_\mathrm{UR2,T} = \sigma_0 + \frac{1-\sigma_0}{1+\left(\frac{T}{C_{0.5}}\right)^{p_\mathrm{UR}}} \quad , \qquad \text{(C7)}$$

$$\sigma_\mathrm{MN2,T} = b(T)^{p_\mathrm{MN}} \quad . \qquad \text{(C8)}$$

Assuming that the contrast responses R_2 and R_1 are proportional to binocular contrast $\hat{C}_2$ and $\hat{C}_1$, based on Eq. 11, the normalized contrast discrimination threshold (by monocular contrast detection threshold) is the solution of the following equation:

$$\hat{C}_2 - \hat{C}_1 = \frac{\sqrt{\sigma_\mathrm{UR1}^2+\sigma_\mathrm{MN1}^2+\sigma_\mathrm{UR2}^2+\sigma_\mathrm{MN2}^2}}{\sqrt{1+\sigma_\mathrm{UR2,T}^2+\sigma_\mathrm{MN2,T}^2}} \quad . \qquad \text{(C9)}$$

aGUM model

With weak pedestal assumption of flank, the alternative GUM (aGUM: Fig. C1) was tested for contrast detection with flank (dotted lines in Figs. 1 and 2). Let w_{F} (Eq. A4) be a flank-target distance weighting function to transfer a flanker to an equivalent weak pedestal. The equivalent weak pedestals of flank F_{L} and F_{R} are given by:

$$Ped_{\mathrm{L}} = w_{\mathrm{F}}(r)F_{\mathrm{L}} \quad \text{and} \quad Ped_{\mathrm{R}} = w_{\mathrm{F}}(r)F_{\mathrm{R}} \qquad \text{(C10)}$$

where r is the flank-target distance in SDU. Taking Eq. C10 into Eqs. C1-C9, we have the predictions of aGUM (dotted lines in Figs. 1-2) for contrast detection thresholds.

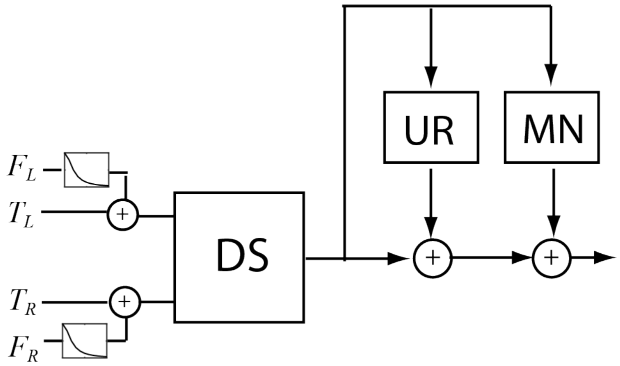

Fig. C1. Alternative GUM (aGUM): wPed model (Fig. A1 A) with UR and MN.

GUMI model

Let $\hat{T}$ be target binocular contrast output of GI model (Fig. 5D), and $\hat{F}_2$ and $\hat{F}_1$ be flank binocular contrast output of GI with and without targets, respectively, which can be calculated by Eq. 6. For a 2AFC task, the internal noise of the interval without target is given by,

$$\sigma_{\mathrm{UR1}} = \sigma_0 + \frac{1-\sigma_0}{1+\left(\frac{w_{\mathrm{N}}\hat{F}_1}{C_{0.5}}\right)^{p_{\mathrm{UR}}}} \quad , \qquad \text{(C11)}$$

$$\sigma_{\mathrm{MN1}} = b(w_{\mathrm{N}}\hat{F}_1)^{p_{\mathrm{MN}}} \quad . \qquad \text{(C12)}$$

and the internal noise of the interval with target is given by,

$$\sigma_{\mathrm{UR2}} = \sigma_0 + \frac{1-\sigma_0}{1+\left(\frac{\hat{T}+w_{\mathrm{N}}\hat{F}_2}{C_{0.5}}\right)^{p_{\mathrm{UR}}}} \quad , \qquad \text{(C13)}$$

$$\sigma_{\mathrm{MN2}} = b(\hat{T} + w_{\mathrm{N}}\hat{F}_2)^{p_{\mathrm{MN}}} \quad , \qquad \text{(C14)}$$

where w_{N} is a flank-target distance weighting function for the internal noise modulated by a flanker, given by,

$$w_{\mathrm{N}}(r) = \frac{1}{1+r^{q_{\mathrm{N}}}} \quad . \qquad \text{(C15)}$$

Assuming that the difference of contrast responses R_2 - R_1 is proportional to target binocular contrast $\hat{T}$, based on Eq. 11, The normalized contrast detection threshold (by monocular target-only threshold) is the solution of the following equation:

$$\hat{T} = \frac{\sqrt{\sigma_{\mathrm{UR1}}^2+\sigma_{\mathrm{MN1}}^2+\sigma_{\mathrm{UR2}}^2+\sigma_{\mathrm{MN2}}^2}}{\sqrt{1+\sigma_{\mathrm{UR2,T}}^2+\sigma_{\mathrm{MN2,T}}^2}} \quad . \qquad \text{(C16)}$$

Where σ_{UR1}, σ_{MN1}, σ_{UR2}, and σ_{MN2} are given by Eqs. C11 – C14, and $\sigma_{\mathrm{UR2,T}}$, and $\sigma_{\mathrm{MN2,T}}$ are given by Eqs. C7 and C8.

Model comparison and best model fits

Table C1 shows fitting statistics of aGUM and GUMI models for data sets of contrast detection shown in Figs. 1 and 2. For contrast discrimination (Figs. 3B and 3C), GUMI is simplified to be GUM without flank-target interactions (flank contrast = 0), and aGUM becomes GUM with flank-target distance = 0. As shown in Table C1, with a weak equivalent pedestal of flank but without separated flank-target interactions, aGUM has poor fitting performance. After including flank-target interactions, GUMI significantly improves fitting performance. Tables C2 – C5 show the best fitting model parameters. Please note that, because the data in Figs. 1-3 are limited, some model parameters are fixed during modeling.

Table C1. Model comparison for each data set

Data	Model	Np	ν	Average			
				χ^2	χ^2/ν	AICc	Likelihood
Fig. 1	aGUM	**6**	43	868.5	20.2	157.6	0.00%
Fig. 1	GUMI	**11**	38	115.9	3.05	74.8	100.00%
Fig. 2AC	aGUM	**4**	8	43.0	5.37	35,3	0.00%
Fig. 2AC	GUMI	**6**	6	0.59	0.10	5.86	100.00%
Fig. 2BD	aGUM	**4**	5	33.8	6.76	41.9	0.00%
Fig. 2BD	GUMI	**3**	6	7.04	1.17	15.8	100.00%
Fig. 3BC	GUM	**5**	10	46.2	4.62	39.4	--

Table C2a. GUMI parameters for fitting data in Fig. 1

Gain-controls & gain-enhancement					Distance weighting functions						
g_{c}	g_{e}	γ	α	β	D_{mfe}	q_{mfe}	D_{ifc}	q_{ifc}	D_{ife}	q_{ife}	q_{N}
1	1	2.55± 0.16	0.85± 0.08	0.46	1	7.31± 0.77	1	4.43± 0.39	3.54± 0.64	4.31± 0.36	0.43± 0.28

Table C2b. GUMI parameters for fitting data in Fig. 1

Uncertainty reduction (UR)			Multiplicative noise (MN)	
σ_0	$C_{0.5}$	q_{UR}	b	q_{MN}
0.35± 0.48	0.44± 0.09	7	0.52± 0.32	0.29± 0.26

Table C3a. GUMI parameters for fitting data in Figs. 2A and 2C

Gain-controls & gain-enhancement					Distance weighting functions						
g_c	g_e	γ	α	β	D_{mfe}	q_{mfe}	D_{ifc}	q_{ifc}	D_{ife}	q_{ife}	q_N
1	1	3.39± 0.62	1	0.46	∞	-	1	2.77	3.99± 0.60	2.79± 0.08	0.95± 0.19

Table C3b. GUMI parameters for fitting data in Figs. 2A and 2C

Uncertainty reduction (UR)			Multiplicative noise (MN)	
σ_0	$C_{0.5}$	q_{UR}	b	q_{MN}
0.35	0.44	7	0.49± 0.07	1.13± 0.14

Table C4a. GUMI parameters for fitting data in Figs. 2B and 2D

Gain-controls & gain-enhancement					Distance weighting functions						
g_c	g_e	γ	α	β	D_{mfe}	q_{mfe}	D_{ifc}	q_{ifc}	D_{ife}	q_{ife}	q_N
1	1	2.11± 0.17	1	0.46	∞	-	1	2.60± 0.07	6.14	2.18	0.95

Table C4b. GUMI parameters for fitting data in Figs. 2B and 2D

Uncertainty reduction (UR)			Multiplicative noise (MN)	
σ_0	$C_{0.5}$	q_{UR}	b	q_{MN}
0.35	0.44	7	0.30± 0.02	1.13

Table C5. GUM parameters for fitting data in Figs. 3B and 3C

Gain-controls		Uncertainty reduction (UR)			Multiplicative noise (MN)	
γ	α	σ_0	$C_{0.5}$	q_{UR}	b	q_{MN}
2.13± 0.48	1.38± 0.13	0	0.52± 0.02	7	0.33± 0.02	0.79± 0.04

References:

1. Ding, J. & Sperling, G. A gain-control theory of binocular combination. *Proceedings of the National Academy of Sciences of the United States of America* **103**, 1141-1146, doi:10.1073/pnas.0509629103 (2006).

2. Ding, J. & Sperling, G. in *Computational Vision In Neural And Machine Systems* (eds L. Harris & M. Jenkin) 257-305 (Cambridge Unversity Press., 2007).

3. Akaike, H. in *Automatic Control, IEEE Transactions.* 716-723 (IEEE).

4. Akaike, H. A new look at the statistical model identification. *Automatic Control, IEEE Transactions on* **19**, 716-723, doi:10.1109/tac.1974.1100705 (1974).
